# Lipid droplets and small extracellular vesicles interplay in Japanese encephalitis virus non-lytic release

**DOI:** 10.1128/mbio.00423-26

**Published:** 2026-05-20

**Authors:** Bhaghyasree Mallick, Ananya Mondal, Ankita Sarkar, Tamoghna Chakraborty, Khadijah Khan, Dilip Kumar, Subhas Chandra Biswas, Sourish Ghosh

**Affiliations:** 1 Infectious Diseases & Immunology Division, CSIR - Indian Institute of Chemical Biology 30156, Kolkata, West Bengal, India; 2 Academy of Scientific and Innovative Research (AcSIR) 550336, Ghaziabad, Uttar Pradesh, India; 3 Cell Biology & Physiology Division, CSIR - Indian Institute of Chemical Biology 30156, Kolkata, West Bengal, India; 4 Trivedi School of Biosciences, Ashoka University 448613, Sonipat, Haryana, India; National Institutes of Health, Bethesda, Maryland, USA

**Keywords:** small extracellular vesicles, lipid droplets, intracellular trafficking, multivesicular bodies, Japanese encephalitis virus, nSMase2, neurons

## Abstract

Lipid droplets (LDs) and small extracellular vesicles (sEVs) are classically known for lipid metabolism and intercellular communication, respectively. Here, we reveal a mechanistic connection between LD dynamics and sEV-mediated non-lytic release of Japanese encephalitis virus (JEV) from neuronal cells. Using Neuro2A, SHSY-5Y, N9 microglia, and primary cortical neurons, we show that JEV is packaged within sEVs (~200 nm) through an ESCRT-independent, neutral sphingomyelinase 2 (nSMase2)/ceramide-dependent pathway. Virions inside sEVs display a higher JEV premature membrane/membrane protein (PrM/M) ratio compared to those released via the conventional secretory pathway. Although containing a higher proportion of premature virions than mature ones, sEV-associated JEV virions gain an evolutionary advantage by evading immune detection and delivering multiple virions to recipient cells, thereby increasing overall infection efficiency. Temporal profiling showed early cytoplasmic LD enrichment (from 6 hpi), followed by a surge in sEV release from 14 hpi, suggesting sequential roles for LDs and sEVs. nSMase2 inhibition decreased sEV-mediated egress without affecting viral replication but increased cytoplasmic LD abundance, consistent with LD underutilization in multivesicular bodies (MVB) biogenesis. Our findings identify LDs as facilitators of MVB formation and nSMase2 as a key driver of sEV-mediated viral exit, revealing parallel yet coordinated pathways in JEV’s stealthy egress.

**IMPORTANCE:**

Lipid droplets and sEVs are traditionally regarded as regulators of lipid homeostasis and intercellular communication. We propose that this LD–sEV connection represents a key mechanism enabling JEV egress from neuronal cells. Japanese encephalitis virus (JEV) exploits small extracellular vesicles (sEVs) for non-lytic viral release from neuronal cells. sEV-containing JEV (~200 nm) is released via an ESCRT-independent, nSMase2/ceramide-dependent pathway. A higher precursor membrane protein (PrM)-to-membrane protein (M) ratio in MVBs and sEVs suggests packaging of immature JEV via a non-secretory pathway. LDs facilitate MVB formation, facilitating sEV-mediated JEV release. sEV release drives LD utilization for MVB formation; nSMase2 knockdown blocks sEV-mediated egress and causes cytoplasmic LD accumulation. JEV enters neuronal cells, releases its RNA from late endosomes, and replicates in the cytoplasm. Virions are subsequently released either through the conventional secretory pathway or by packaging into multivesicular bodies (MVBs) and secretion within small extracellular vesicles (sEVs). Maturation of JEV requires cleavage of the premature membrane protein (PrM) into the membrane protein (M). Notably, the PrM/M ratio is higher in virions released via the secretory pathway compared to those packaged within sEVs, where a greater proportion of immature virions are enclosed. Lipid droplets (LDs), derived from the endoplasmic reticulum (ER), interact with MVBs and contribute to JEV release inside sEVs. This process is driven by a ceramide-dependent, ESCRT-independent pathway regulated by nSMase2. Together, the model highlights the coordinated role of the LD–sEV axis in mediating JEV non-lytic egress.

## INTRODUCTION

Host-pathogen interaction is influenced not only by the immune system but also by a tightly coordinated network of membrane-associated organelles that control cargo storage, trafficking, and metabolic balance (1). Among the most intriguing of these are small extracellular vesicles (sEVs)—once considered trivial cellular by-products, now recognized as powerful modulators of intercellular communication, metabolic signaling, and viral trafficking (2–5). Extracellular vesicles (EVs), including exosomes and microvesicles, are nanoscale, lipid bilayer-enclosed carriers secreted by nearly all cell types with particle sizes typically ranging from 30 to 1,000 nm (6). Generated either by ESCRT-dependent machinery or ESCRT-independent pathways, EVs transport bioactive molecules such as proteins, RNA, and lipids across cells and tissues, shaping immune responses, tissue remodeling, and—most pertinently—viral spread (7–9). sEVs have emerged as important mediators of intercellular communication and have been implicated in the spread of infection in several flaviviral models, such as Dengue virus (DENV), Zika virus (ZIKV), and West Nile virus (WNV), including Japanese encephalitis virus (JEV) (10–15). Neutral sphingomyelinase 2 (nSMase2), also known as SMPD3, has been shown to regulate the biogenesis and release of sEVs in neurons (16, 17). Inhibition of SMPD3, either through siRNA-mediated knockdown or pharmacological blockade using GW4869, resulted in reduced ZIKV loads in neurons and neuron-derived extracellular vesicles (sEVs) (18). A similar observation was made in the case of JEV, where GW4869 inhibited both replication and virus release inside sEVs in various cell types, emphasizing the role of the nSMase2-ceramide pathway in EV-mediated flaviviral transmission (13).

In contrast to extracellular vesicles, lipid droplets (LDs) are intracellular organelles that serve as energy reservoirs, consisting primarily of neutral lipids such as triacylglycerols (TAGs) and cholesteryl esters, encased within a phospholipid monolayer (19). LDs have evolved from mere lipid stores into dynamic, multifunctional platforms that play a role in protein sequestration, immune signaling, lipid trafficking, and viral replication, including flaviviruses (20–24). Pharmacological inhibition of DGAT-1, an enzyme involved in LD biogenesis, has been shown to significantly reduce LD formation and ZIKV replication in both *in vitro* and *in vivo* systems (25). Beyond their role in viral replication, LDs also contribute to the early antiviral immune response, particularly through their influence on type I interferon (IFN) signaling following infection, thereby helping to control viral spread (20). Consistent with the immunomodulatory functions of LDs, blockade of LD formation also profoundly affects inflammatory cytokine production in the brain, highlighting the dual role of LDs in both viral pathogenesis and host immune regulation (26).

While the individual roles of EVs and LDs have been extensively studied in the context of viral infections, their interconnection has not been thoroughly explored. A recent study revealed that an abundance of LDs strongly correlates with the release of sEVs in various cancer cell lines (27). Pharmacological inhibition of LD metabolism significantly suppressed sEV secretion, while metabolic stressors such as hypoxia, acidosis, or irradiation—known to enhance LD biogenesis—dramatically elevated vesicle release. These findings suggest that lipid metabolic flux may govern the dynamics of EV biogenesis. Further linking these organelles is the ceramide pathway, a shared molecular axis involving neutral sphingomyelinase 2 (nSMase2), a key enzyme that converts sphingomyelin into ceramide—a lipid critical for both EV budding and LD turnover (28, 29). Notably, nSMase2 is highly expressed in the central nervous system (CNS) and plays essential roles in brain development, membrane remodeling, and neuronal communication. Viruses, including flaviviruses, are known to hijack the ESCRT-independent, ceramide-dependent route to enhance their transmission (30).

JEV is a mosquito-borne, neurotropic flavivirus and the leading cause of viral encephalitis in Asia and the Western Pacific (31). Globally, more than 68,000 cases of Japanese encephalitis (JE) are reported annually, with a case fatality rate approaching 30%. Among survivors, 20–30% suffer long-term neurological sequelae, including motor impairments, recurrent seizures, and cognitive or speech deficits (32). Although significant progress has been made in understanding JEV pathogenesis and host-pathogen interactions, the mechanisms underlying viral egress, particularly from neuronal cells, remain poorly defined. Recent studies suggest that JEV can exit host cells encapsulated in sEVs, but the molecular determinants of this process remain unclear. Given the emerging recognition of LDs and EVs as central hubs in lipid metabolism and intercellular signaling, we sought to determine whether JEV exploits the LD–sEV axis to facilitate its release from neuronal and glial cells, with a specific focus on the ESCRT-independent pathway mediated by the ceramide-producing enzyme nSMase2.

## RESULTS

### JEV egress from neuronal cells via a non-lytic mechanism

**Fig 1:**
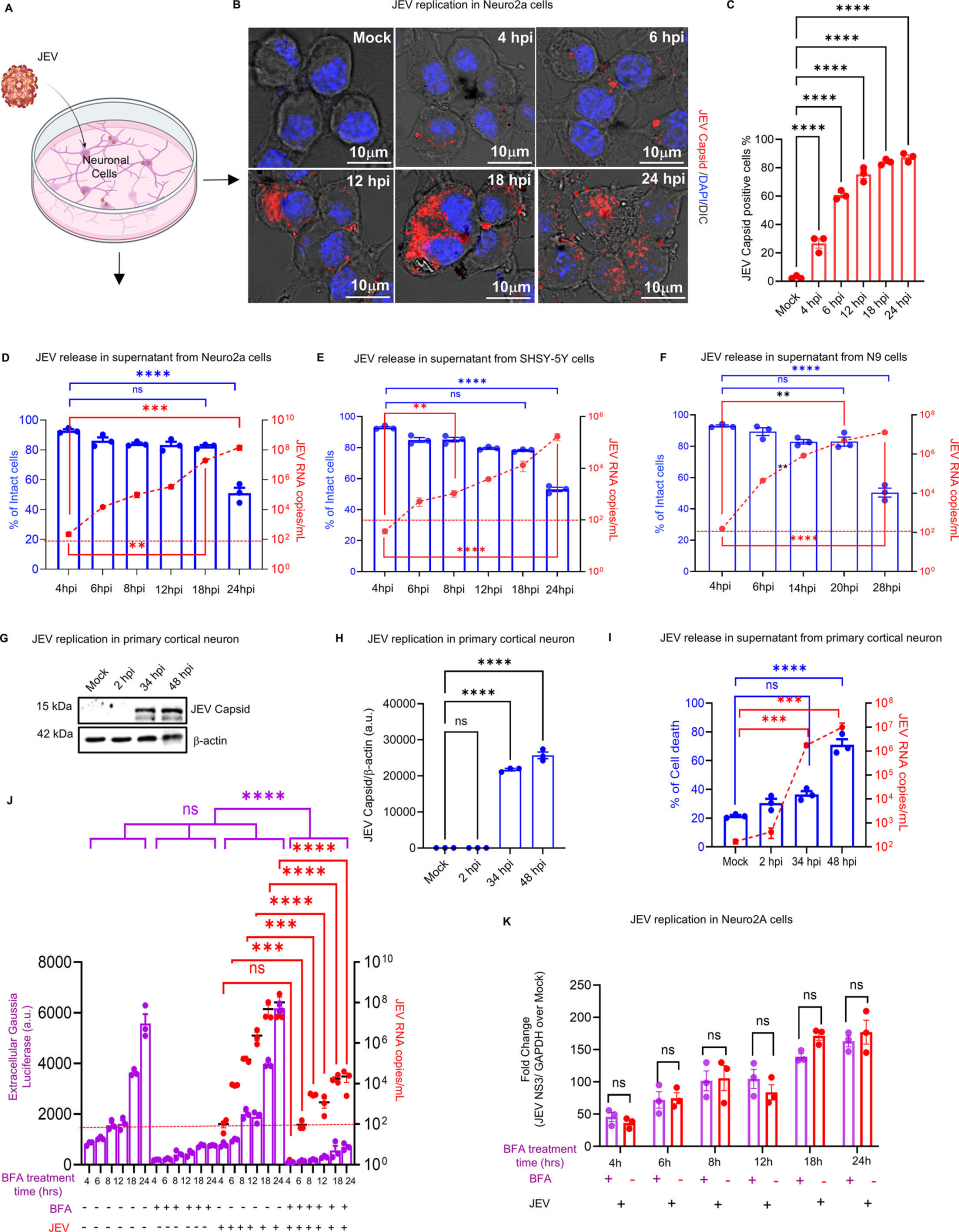
JEV egress from neuronal cells via a non-lytic mechanism. (**A**) Neuronal cells were inoculated with JEV to determine optimal time points for egress analysis. (**B**) Representative immunostaining images showing JEV capsid protein expression in mock and JEV-inoculated Neuro2a cells at 4, 6, 12, 18, and 24 hpi. (**C**) Percentage of JEV capsid-positive cells over the total number of cells in the field was quantified. Each data point represents the percentage of JEV capsid-positive cells per frame. Scale bar: 10 µm. Experiments were performed in triplicates. (**D–F**) JEV RNA copies were measured in the culture supernatants collected at various time points from (**D**) Neuro2a, (**E**) SHSY-5Y, and (**F**) N9 microglial cell lines. Corresponding cell viability was assessed using trypan blue exclusion. The red line represents the limit of detection for JEV RNA copies by RT-qPCR. Experiments were done in triplicates. (**G**) Representative Western blot image showing JEV capsid protein expression in Mock and JEV-inoculated primary cortical neurons at 2, 34, and 48 hpi. JEV capsid protein expression was quantified by densitometry (**H**), with β-actin as the loading control. Experiments were done in triplicates. (**I**) JEV RNA copies in the supernatant of mock- and JEV-infected primary cortical neurons at 2, 34, and 48 hpi were quantified by RT-qPCR, and corresponding cell viability was assessed by trypan blue exclusion. Experiments were done in triplicates. (**J**) Neuro2a cells were treated without and with BFA (5 µg/mL) for 4, 6, 8, 12, 18, or 24 h prior to the collection of extracellular medium at 24 hpi. JEV egress and Gaussia luciferase secretion were quantified from the collected supernatants. All experiments were performed in triplicates. (**K**) Quantitative analysis of JEV-NS3 mRNA expression levels from cell lysate at different time points with or without BFA. All experiments were performed in triplicates. Statistical significance was assessed using unpaired *t*-tests for pairwise comparisons and one-way ANOVA followed by multiple comparison tests for multiple comparisons. *P < 0.05, **P < 0.01, ***P < 0.001, ****P < 0.0001; ns, not significant.

JEV, a well-known neurotropic virus, efficiently infects various neuronal cell lines, including Neuro2a, SHSY-5Y, and N9 microglial cells, as well as primary cortical neurons (33, 34). These models were examined for JEV replication and release (Fig. 1A). To characterize the kinetics of JEV replication in Neuro2a cells, we monitored capsid protein expression and intracellular infectious virus production over 24 h (Fig. 1B; Fig. S1A and B). JEV capsid protein was detected as early as 6 hpi, marking the onset of viral replication (Fig. 1B; Fig. S1A). The proportion of capsid-positive cells increased to about 80% between 18 and 24 hpi, along with the increase in capsid signal intensity, indicating viral replication inside the cells (Fig. 1C). To assess JEV egress, JEV RNA copy numbers in the supernatant of infected Neuro2a, SHSY-5Y, and N9 cells were quantified. A significant increase in extracellular JEV RNA copies was detected at 18 hpi in Neuro2a and SHSY-5Y cells and at 20 hpi in N9 cells (Fig. 1D through F). Consistent with these findings, measurement of infectious viral titers in the culture supernatant showed a significant increase at 18 hpi in Neuro2a and SHSY-5Y cells and at 20 hpi in N9 cells (Fig. S1E through G). Notably, cell viability remained largely unaffected during this period, as determined by the trypan blue exclusion assay, indicating that viral release occurred without substantial cell lysis. Further, we infected primary cortical neurons with JEV. Robust viral replication was evident from capsid protein and JEV non-structural NS3 mRNA expression at 34 and 48 hpi (Fig. 1G and H; Fig. S1C), while cortical neurons remained morphologically intact up to 34 hpi and non-lytically released JEV (Fig. 1I; Fig. S1D).

JEV uses the classical biosynthetic secretory pathway for egress. To examine that, cells were transfected with a Gaussia luciferase plasmid and then infected with JEV. Luciferase secretion kinetics remained largely unchanged during infection, indicating that the secretory pathway stayed functional (Fig. 1J). To test pathway dependence, cells were treated with Brefeldin A (BFA; 5 μg/mL) (Fig. S1H) at 2, 6, 12, 16, 18, or 20 h post-infection (hpi), and supernatants were collected at 24 hpi (4–24 h of BFA exposure) (Fig. 1J). BFA, an inhibitor of ER–Golgi transport that induces Golgi collapse (35, 36), effectively blocked luciferase secretion. Although BFA treatment did not affect intracellular JEV replication (Fig. 1K), it significantly reduced extracellular viral genome release (Fig. 1J). However, detectable virus remained in the supernatant, suggesting that while the ER–Golgi pathway contributes to JEV egress, JEV can also exit via alternative routes, such as extracellular vesicle–mediated secretion.

### JEV is released via small extracellular vesicles (sEVs)

**Fig 2:**
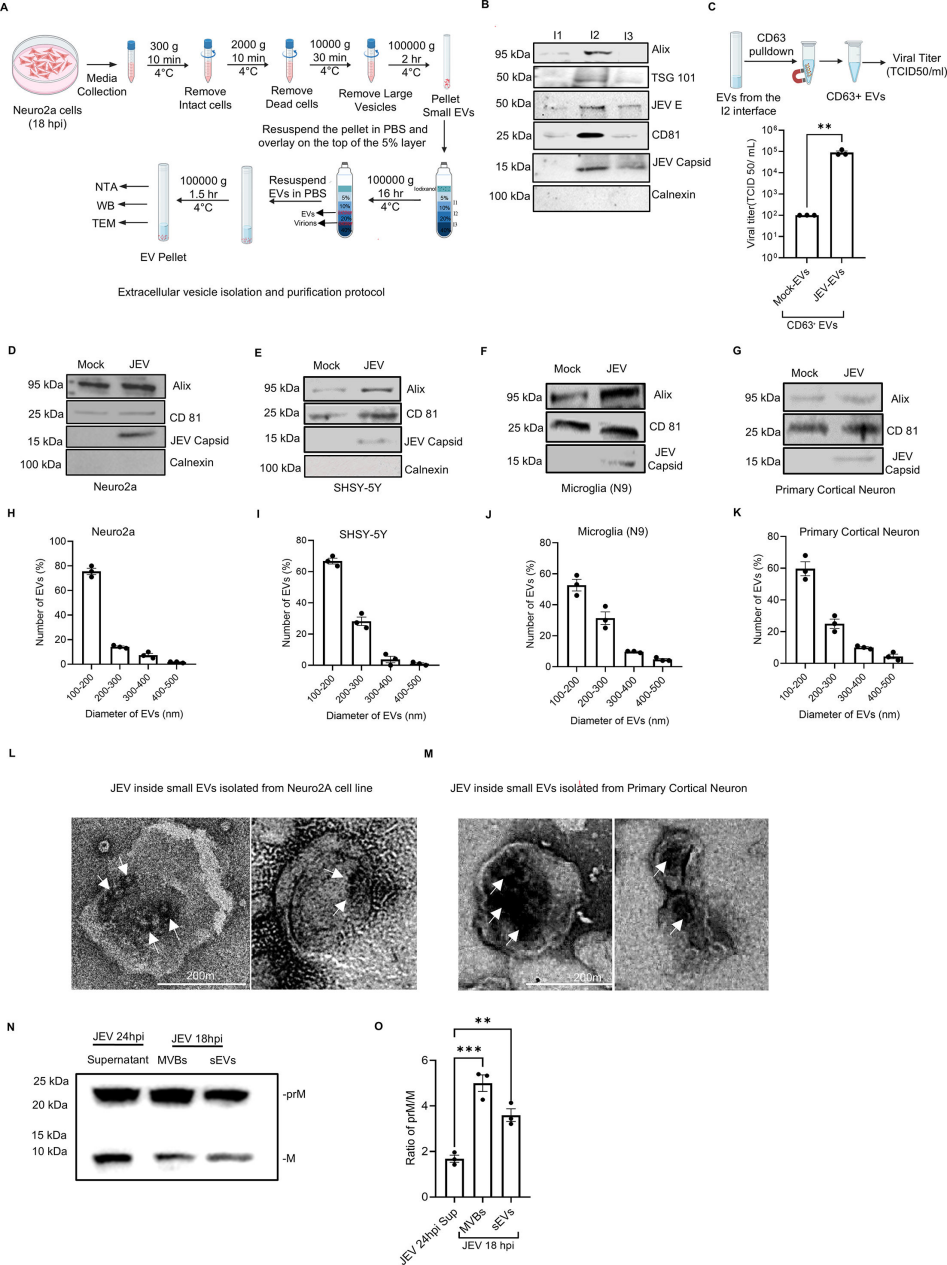
JEV is released via small extracellular vesicles (sEVs). (**A**) A schematic outlines the workflow used to isolate and enrich small extracellular vesicles (sEVs) from cell culture supernatants. (**B**) Representative Western blot showing the protein expression of Alix, CD81, and TSG 101, along with JEV capsid and JEV E protein from I1, I2, and I3 interface shown in panel **A**. Calnexin was used as a negative control to assess organellar contamination. Equal volume of each sample was loaded to ensure normalization. Experiments were performed in triplicates. (**C**) CD63-positive EVs were isolated from the I2 interface using anti-CD63 antibody–conjugated protein A/G magnetic beads, and viral titer was quantified by TCID50/mL. Experiments were done in triplicates. (**D–G**) Representative Western blot showing protein expression of EV markers Alix and CD81 and JEV capsid in mock- and JEV-infected Neuro2a (**D**), SHSY-5Y (**E**), N9 microglial cells (**F**), and primary cortical neurons (**G**) isolated sEVs. Calnexin was included as a negative control in Neuro2a and SHSY-5Y sEVs (as mentioned in panel B). Equal volumes of each EV preparation were loaded to ensure normalization. Experiments were done in triplicates. (**H–K**) The size distribution of vesicles was measured using NTA in Neuro2a (**H**), SHSY-5Y (**I**), microglia (**J**), and primary cortical neurons (**K**). Experiments were done in triplicates. (**L and M**) Transmission electron microscopy (TEM) with negative staining visualized sEVs carrying JEV particles in (**L**) Neuro2a cells and (**M**) primary cortical neurons. Arrows indicate the presence of JEV particles inside the vesicles. Scale bar = 200 nm. (**N and O**) Measuring cleavage of the premature membrane protein (prM) into the mature membrane protein (M) of JEV in cell lysate, multi-vesicular bodies (MVBs), and small extracellular vesicles (sEVs) collected at 24 hpi and 18 hpi, respectively. (**O**) Densitometric quantification of the prM/M ratio across the indicated groups. An equal volume of samples was added to wells for normalization. Experiments were done in triplicates. Statistical significance was assessed using unpaired *t*-tests for pairwise comparisons and one-way ANOVA followed by multiple comparison test for multiple comparisons. **P < 0.01, ***P < 0.001.

To investigate whether JEV utilizes EVs for its release, supernatants from cells were processed using a sequential centrifugation protocol as illustrated in (Fig. 2A). We isolated EVs from Neuro2a by differential ultracentrifugation followed by iodixanol-mediated density-gradient centrifugation, and three interfaces (I1, I2, and I3) were collected (Fig. 2A; Fig. S2A). I1 contained lighter particles and debris, and I2 contained the EV-enriched fraction, whereas I3 predominantly contained EV-free virions. Western blot analysis revealed that EV markers, Alix, CD81, and TSG101 are majorly expressed in I2 compared to I1 and I3, indicating that I2 indeed comprised bona fide EVs devoid of calnexin (endoplasmic reticulum [ER] marker) (Fig. 2B), validating the collection of pure EVs. The presence of JEV E and capsid protein in I2 and I3 indicates that both interfaces contain the virus inside EVs or as free particles. To further validate that I2 contained the virus inside vesicles and not as free particles, CD63-positive EVs (CD63, EV marker) were purified using anti-CD63 antibody–conjugated magnetic beads from the I2 interface obtained after iodixanol density gradient centrifugation; viral titer was measured by TCID50/mL (Fig. 2C). Nanoparticle tracking analysis (NTA) confirmed the presence and size distribution of CD63 A/G bead–pulldown vesicles (Fig. S2B).

To confirm JEV egress inside EVs, vesicles were isolated from JEV-infected Neuro2a (Fig. 2D), SHSY-5Y (Fig. 2E), N9 microglial cells (Fig. 2F), and primary cortical neurons (Fig. 2G) and analyzed for the presence of extracellular vesicle markers Alix and CD81, together with JEV capsid protein. Western blot analysis showed significant increase in all three proteins in JEV-infected samples compared with mock controls (Fig. S2C through E). An equal volume of each EV preparation was loaded to ensure normalization across samples. To determine the size distribution of EVs, NTA was performed. The diameters of purified vesicles ranged from 100 to 500 nm across all four cell types (Fig. 1H through K), with the majority of EVs falling within the 100–200 nm size range of sEVs. Additionally, ultrastructural validation was performed using transmission electron microscopy (TEM) imaging with negative staining. Vesicles isolated from both Neuro2a (Fig. 2L; showing an opened vesicle on the left, while the right one is intact) and primary cortical neurons (Fig. 2M) were observed to contain 2–3 JEV particles per vesicle. Aligning with these observations that JEV virions are assembled at the ER, do not pass through the normal biosynthetic pathway, and are packaged inside EVs, a key question arises: whether these JEV virions are in their mature or immature state. Furin, a Golgi-resident host enzyme, cleaves JEV premature membrane protein (PrM) to membrane protein (M), exposing the E protein and facilitating host receptor attachment, resulting in mature viral particles. The PrM/M ratio signifies the infectivity of JEV (37): the lesser the ratio, the more the infectivity. Our results show that the PrM/M ratio in JEV-infected cell supernatant at 24 hpi was lower than that in MVBs (isolated by subcellular fractionation and confirmed for fraction purity as detailed in Materials and Methods) (Fig. S2F) and in sEVs collected at 18 hpi, indicating a higher proportion of immature viral particles packaged within MVBs and sEVs (Fig. 2N and O). Hence, immature virions are being packaged inside sEVs, providing a faster route of egress bypassing the ERGIC compartment. Overall, the data support that JEV is packaged and secreted within sEVs across multiple neuronal cell types. However, it remained unclear whether immature JEV virions enclosed within vesicles are infectious.

### JEVs packaged within sEVs are infectious

**Fig 3:**
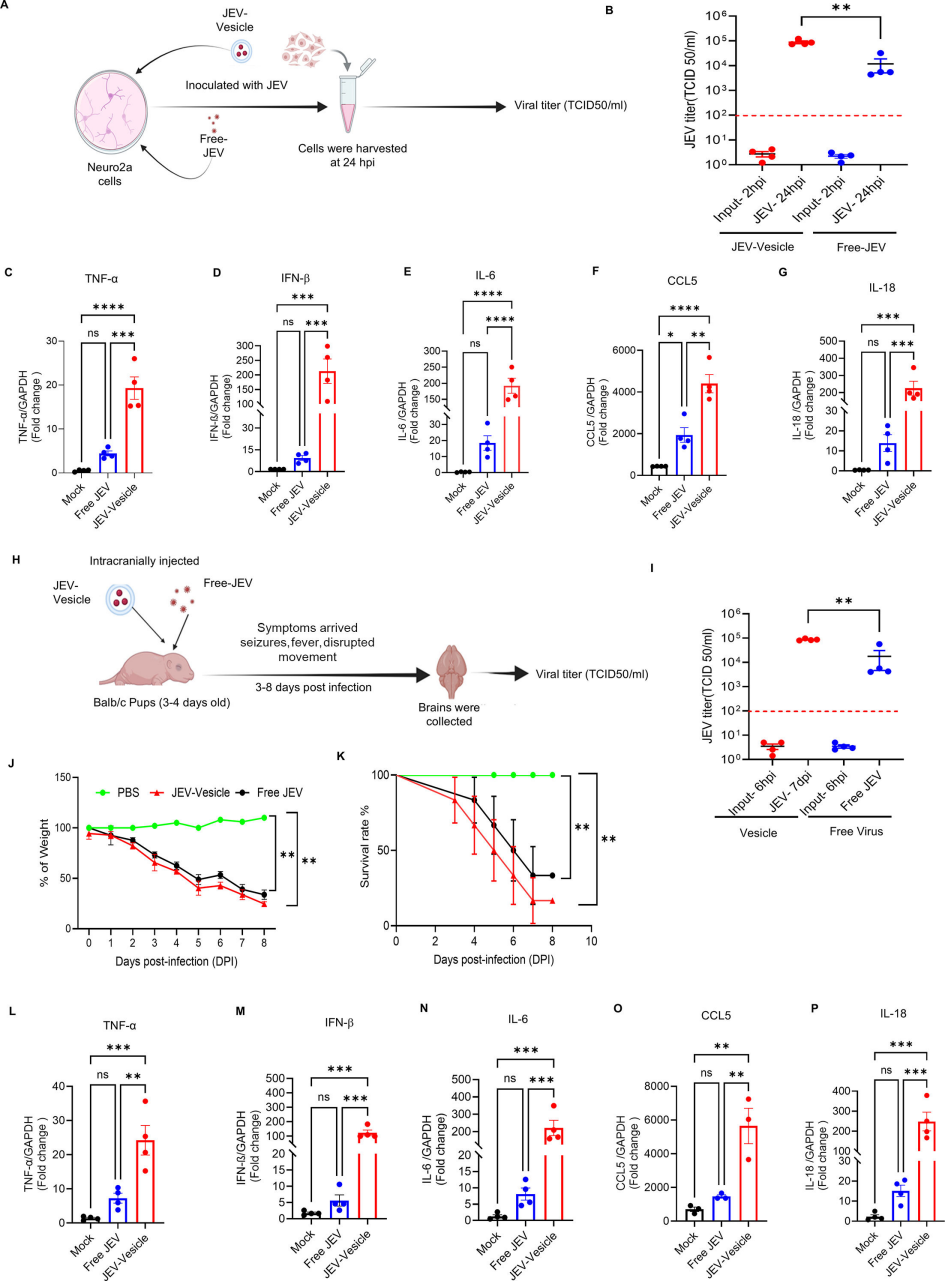
JEV packaged within sEVs are infectious. (**A and B**) Neuro2a cells were inoculated with Neuro2a cell–derived sEV-JEV or free JEV containing equal JEV RNA copy numbers (based on equal inoculation volume) (**A**). Cells from each group were harvested at the indicated time points, and viral titers were quantified (TCID50/mL) (**B**). Red line represents the limit of detection for TCID50/mL. Experiments were done in triplicates. (**C–G**) mRNA levels of TNF-α (**C**), IFN-β (**D**), IL-6 (**E**), CCL5 (**F**), and IL-18 (**G**) in cell lysates were quantified as fold change for free JEV and JEV vesicle over mock. Experiments were done in triplicates. (**H**) Four-day-old BALB/c mouse pups were intracranially injected with JEV vesicle or free JEV containing equal JEV RNA copy numbers (based on equal inoculation volume). (**I**) Brain tissues were harvested at 7 dpi, and viral titers were measured by TCID50 assay. Red line represents the limit of detection for TCID50/mL. Experiments were done in triplicates. (**J**) % of weight loss and (**K**) survival rate (%) were compared between groups receiving JEV vesicle versus free JEV. (**L–P**) mRNA levels of TNF-α (**L**), IFN-β (**M**), IL-6 (**N**), CCL5 (**O**), and IL-18 (**P**) in brain tissues were quantified between groups JEV vesicle versus free JEV by RT-qPCR. Experiments were done in triplicates. Statistical significance was assessed using unpaired *t*-tests for pairwise comparisons and one-way ANOVA followed by multiple comparison tests for multiple comparisons.*P < 0.05, **P  <  0.01, ***P  <  0.001, ****P  <  0.0001; ns not significant.

We compared the relative infectivity of JEV released via sEVs versus free virus particles obtained from tsEVs. JEV-containing sEVs were isolated and divided into two fractions: one left intact and the other lysed using a detergent-free lysis buffer (Fig. S2G). To confirm that the lysis buffer did not impair viral infectivity, an equal amount of JEV stock was treated with the same buffer, and its replication capacity was compared with untreated virus in Neuro2a cells (Fig. S2H and I). JEV titers (TCID50/mL) and RNA copy numbers were measured for equal volume of intact JEV-containing vesicles and free JEV particles released after lysis (Fig. S2J), and no significant difference in viral load was observed. Next, an equivalent volume of either intact sEV-associated JEV or vesicle-free JEV (lysed fraction) was inoculated onto Neuro2a cells (Fig. 3A). At 24 hpi, cells infected with JEV cloaked inside sEVs yielded significantly higher titers compared to those infected with EV-free virus (Fig. 3B), indicating superior infectivity of the vesicle-associated form. Inflammation is one of the signatures of JEV infection; hence, we measured the cytokine levels in cells after inoculating with these two forms of JEV—within sEVs and as free particles. Elevated expression of TNF-α, IFN-β, IL-6, CCL5, and IL-18 was observed in cells inoculated with JEV-EVs compared with those infected with EV-free virions (Fig. 3C through G). To further evaluate this difference in infectivity, 4-day-old BALB/c mouse pups—an established model for JEV infection—were intracranially inoculated with either form of the virus (Fig. 3H). This route bypasses the blood-brain barrier and ensures equal amounts of virus (both forms) delivery to the brain. At 7 days post-inoculation, brain tissues from the group inoculated with sEV-associated JEV showed markedly higher viral titers than those inoculated with free virus particles (Fig. 3I). Moreover, mice receiving the sEV-associated JEV exhibited more rapid and pronounced weight loss (Fig. 3J) and succumbed to infection earlier (Fig. 3K), consistent with a more severe disease phenotype. Similar to the *in vitro* analysis, the same cytokines showed elevated levels of expression from brains intracranially injected with sEV containing JEV (Fig. 3L through P). Together, these results suggest that sEV-mediated transmission of JEV, even having a higher proportion of immature virions to mature virions, is infectious. It is hypothesized that cleavage of PrM to M exposes the E protein, helping in JEV attachment to host receptors for entry; however, vesicle-cloaked virions can bypass the requirement by getting packaged inside sEVs. How sEVs enter cells while carrying the virions and delivering them to new hosts needs further investigation.

### JEV-induced sEV release occurs via a ceramide-dependent, ESCRT-independent pathway

**Fig 4:**
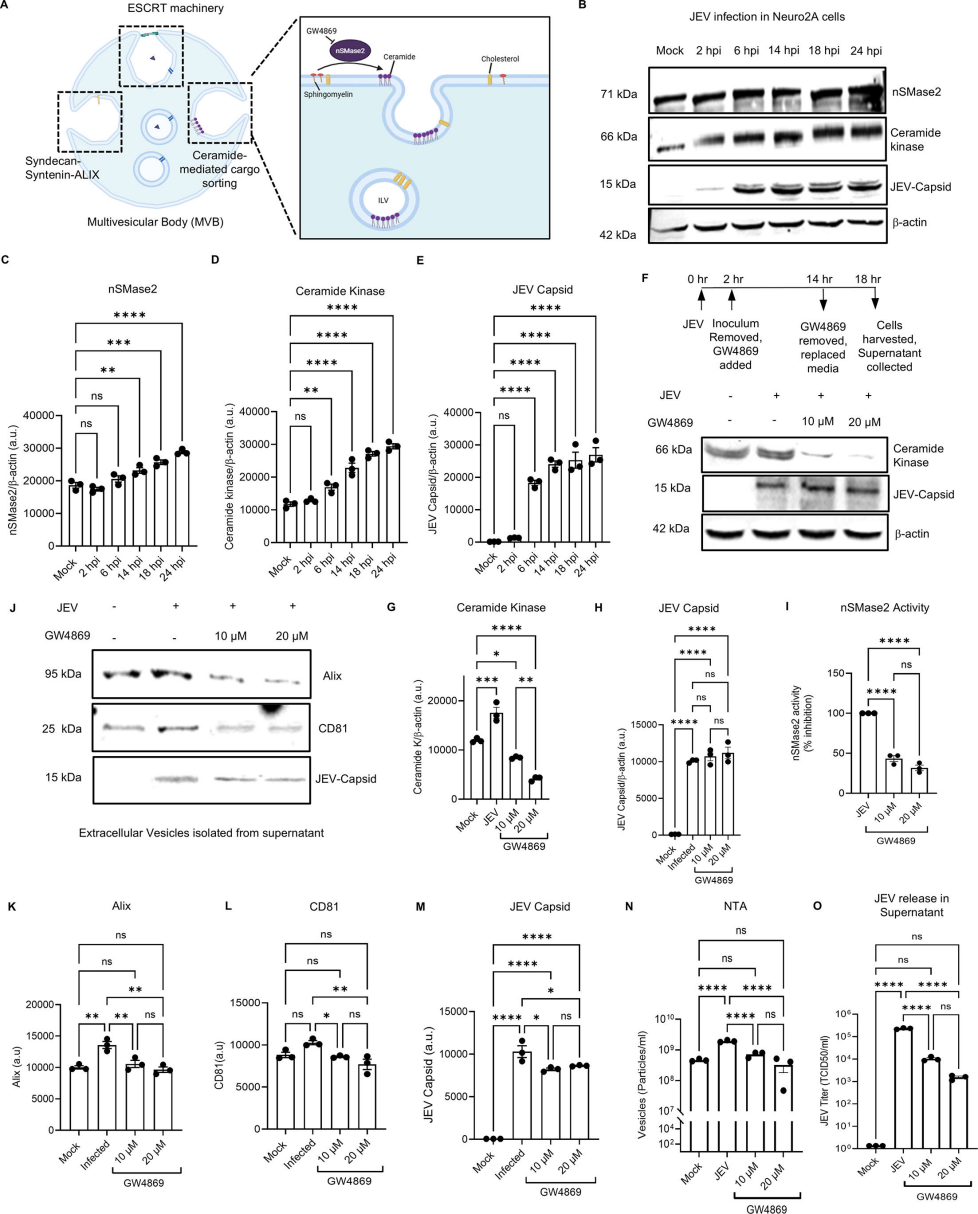
JEV-induced sEV release occurs via a ceramide-dependent, ESCRT-independent pathway. (**A**) Schematic representation of ESCRT-dependent and independent pathways of cargo sorting. In the ceramide-mediated cargo sorting, sphingomyelin is converted to ceramide by the enzyme nSMase2. Ceramide contributes to the formation of curvature on MVBs for intraluminal vesicle (ILV) formation. GW4869 inhibits nSMase2 activity. (**B**) Representative Western blot showing nSMase2 and ceramide kinase protein levels, alongside JEV capsid protein expression at different time points. β-actin was used as a loading control. (**C–E**) Densitometric quantification of nSMase2 (**C**), ceramide kinase (**D**), and JEV capsid protein (**E**) levels from panel B. Experiments were done in triplicates. (**F**) Representative Western blot showing expression of ceramide kinase and JEV capsid protein after treatment with GW4869 (10 µM and 20 µM for 12 h) followed by harvesting cells infected with JEV at 18 hpi and (**G and H**) corresponding densitometric analyses. β-actin was used as a loading control. Experiments were done in triplicates. (**I**) nSMase2 activity was measured after treatment with GW4869 (10 or 20 µM) for 12 h and expressed as percentage inhibition compared to only JEV-inoculated cells. All experiments were performed in triplicate. From the same wells, supernatants were collected, and sEVs were isolated. (**J**) Representative Western blot showing expression of vesicle markers Alix and CD81 along with JEV capsid protein in isolated sEVs and (**K–M**) their densitometric quantification. An equal volume of samples was added to wells for normalization. Experiments were done in triplicates. (**N**) Vesicle concentration (particles/mL) was measured using NTA, and (**O**) JEV titer (TCID50/mL) was determined from the same samples. Experiments were done in triplicates. Statistical analysis was performed using one-way ANOVA with multiple comparisons. *P < 0.05, **P  <  0.01, ***P  <  0.001, ****P  <  0.0001; ns not significant.

To investigate the mechanism of sEV release following JEV replication within neuronal cells, we first studied the ESCRT-dependent pathway. VPS4B (an essential AAA ATPase of the ESCRT-dependent pathway) expression was silenced in Neuro2a cells using siRNA. Representative immunoblotting confirmed efficient knockdown of VPS4B and showed a corresponding reduction in JEV capsid protein levels from cell lysate (signifying JEV replication) following VPS4B siRNA treatment (Fig. S3F through H). Since JEV replication was interfered with due to VPS4B knockdown, both viral titer (Fig. S3I) and JEV NS3 mRNA expression from cell lysate (Fig. S3J) were perturbed. Sphingomyelin (SM), a key membrane lipid enriched in neuronal cells, is enzymatically converted to ceramide by neutral sphingomyelinase 2 (nSMase2)—a well-characterized mechanism involved in the biogenesis of multivesicular bodies (MVBs) and sEVs (38–40). Given the neurotropism of JEV, we investigated whether this ceramide-driven, ESCRT-independent pathway facilitates viral release via sEVs during infection (schematic, Fig. 4A). Immunoblot analysis revealed a time-dependent upregulation of nSMase2 and ceramide kinase expression in Neuro2a cells inoculated with JEV from 6 to 24 hpi, along with increased viral capsid expression (Fig. 4B through E). This suggests that JEV infection actively engages the ceramide pathway during replication. To functionally validate this mechanism, we employed GW4869—a pharmacological inhibitor of nSMase2 previously shown to block ceramide-dependent vesicle biogenesis (41). Neuro2a cells were treated with optimized concentrations of GW4869 (10–20 µM), as determined by MTT assay (Fig. S3A), and a schematic overview of the experimental design is shown in Fig. S3D. Following JEV infection, cells were treated with GW4869 (10 µM or 20 µM) for different durations (2, 6, or 12 h), after which samples were collected for analysis. Viral titer measurements showed that 12 h of GW4869 treatment significantly reduced viral release into the cell supernatant. This treatment also markedly decreased ceramide kinase expression without impairing intracellular JEV replication, as confirmed by Western blotting (Fig. 4F through H) and JEV NS3 mRNA quantification in cell lysates collected at 18 hpi using GAPDH as a housekeeping control (Fig. S3B). Neuro2a cells treated with GW4869 showed a significant reduction in nSMase2 activity at 10 µM and 20 µM concentrations compared to untreated JEV-infected control cells (Fig. 4I), confirming the effectiveness of GW4869 under these experimental conditions and supporting its role in inhibiting ceramide-dependent extracellular vesicle biogenesis. To assess the infectivity of released virus, Neuro2a cells were inoculated with cell-free supernatants from GW4869-treated cultures. Viral infectivity was quantified as TCID50/mL, revealing reduced infectivity compared with untreated infected controls (Fig. S3C). Together, these findings indicate that inhibition of the ceramide pathway suppresses vesicle-mediated viral release without causing cytotoxicity or interfering with viral genome replication.

sEVs isolated from GW4869-treated cells supernatant displayed significantly reduced levels of canonical vesicle markers Alix and CD81, as well as JEV capsid protein (Fig. 4J through M). This was further supported by NTA, which revealed a significant reduction in vesicle concentration (Fig. 4N), and by decreased JEV titers measured from the vesicle fractions (Fig. 4O). Collectively, these data demonstrate that JEV utilizes a ceramide-mediated, ESCRT-independent pathway for packaging and release via sEVs. Pharmacological blockade of this pathway significantly attenuates the release of virus-loaded vesicles without affecting intracellular replication, underscoring the specificity and functional importance of this egress mechanism.

### JEV infection regulates lipid droplet dynamics to facilitate viral egress via a ceramide-mediated cargo sorting mechanism

**Fig 5:**
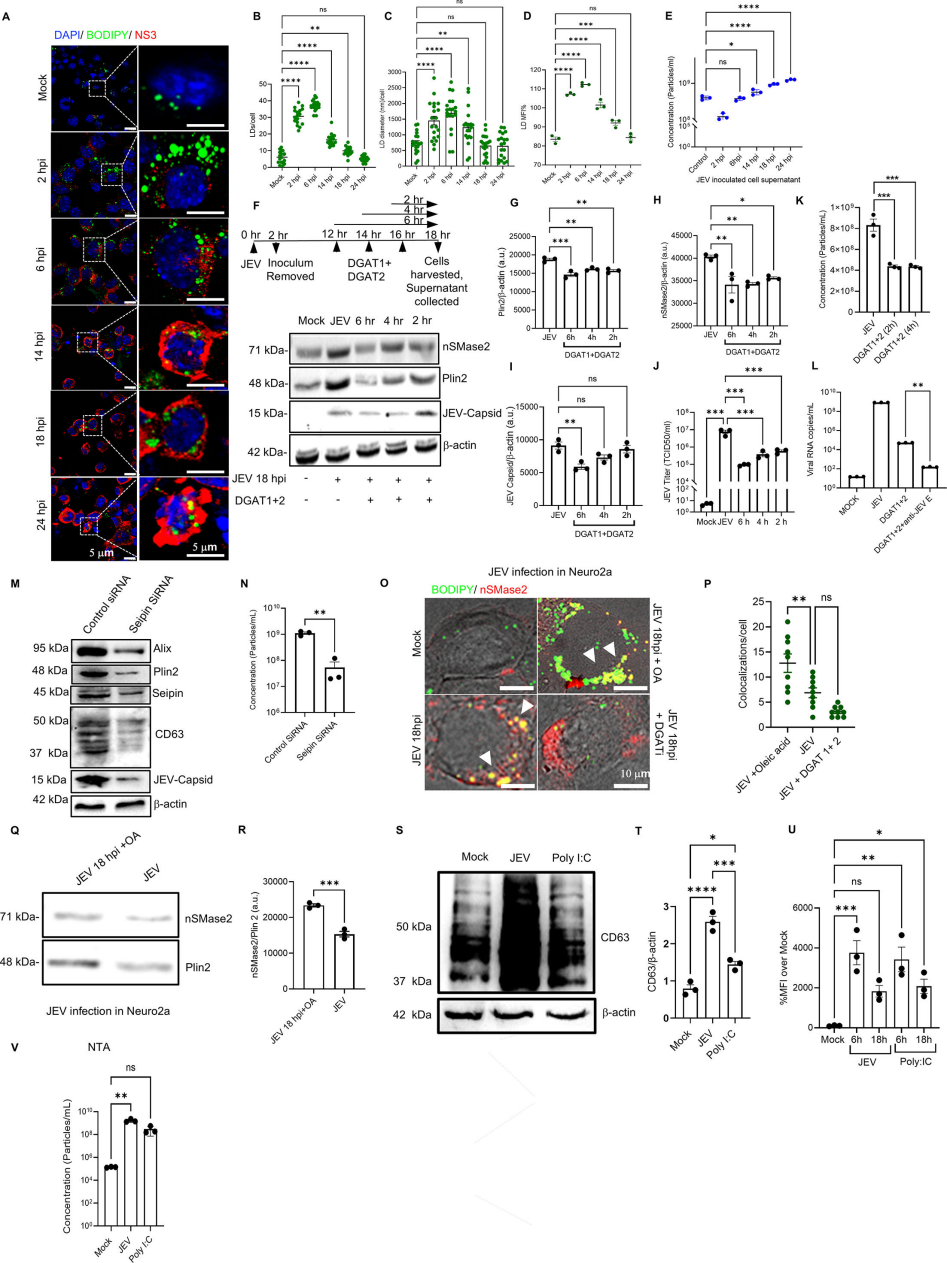
JEV infection regulates lipid droplet (LD) secretion to facilitate viral egress via ceramide-mediated cargo sorting mechanism. (**A**) Representative immunostaining images showing BODIPY-stained LD and JEV-NS3 expression at 2, 6, 14, 18, and 24 hpi. Enlarged views of selected cells are shown. Scale bar = 7.5 µm. (**B**) Quantification of LD number per cell at the indicated time points. Each data point represents an individual cell. (*n* = 20 per group). (**C**) Quantification of LD size (*n* = 20 per group). Each data point represents a single LD from one cell in a field of view. (**D**) Flow cytometric analysis of BODIPY-stained LDs at the indicated time points showing mean fluorescence intensity percentage (MFI%). (**E**) Supernatants collected from the same wells were used to isolate sEVs, and their concentrations (particles/mL) were quantified by NTA. Experiments were done in triplicates. (**F**) Neuro2a cells were treated with DGAT1 + 2 inhibitors for 2, 4, or 6 h during JEV infection and analyzed by Western blot for nSMase2, Plin2, and JEV-capsid. β-actin was the loading control. (G–I) Densitometric quantification of (**G**) Plin2, (**H**) nSMase2, and (**I**) JEV capsid protein from Western blots shown in panel F. (**J**) JEV titers (TCID50/mL) were measured from sEVs isolated from the same treatment wells. (**K**) sEVs isolated from JEV-infected Neuro2a cells after DGAT inhibitor treatment for 2 or 4 h were analyzed by NTA at 18 hpi. Experiments were done in triplicates. (**L**) JEV RNA copy numbers were measured in supernatants from mock- and JEV-infected cells (18 hpi) with DGAT1 + 2 inhibitors alone or in combination with anti-JEV-E protein. Experiments were done in triplicates. (**M and N**) Representative Western blot showing Alix, CD63, Seipin, Plin2, and JEV capsid protein expression in control siRNA and Seipin siRNA–transfected cells following JEV infection (18 hpi). β-actin was used as a loading control. EV concentrations in corresponding supernatants were measured by NTA (**N**). All experiments were performed in triplicates. (**O**) Representative immunostaining images showing colocalization of LD and nSMase2 under the following conditions: Mock, JEV (18 hpi) + oleic acid (OA), JEV infection (18 hpi), and JEV (18 hpi) + DGATi (2h). Arrowheads indicate regions of LD–nSMase2 colocalization. Scale bar = 10  µm. Representative images from one out of three biologically independent experiments were shown. (**P**) Quantification of LD–nSMase2 colocalizations per cell under the indicated conditions. Each data point represents a single cell. (**Q and R**) Representative Western blot showing nSMase2 levels in LDs isolated from JEV-infected cells (18 hpi) with or without OA treatment, using Plin2 as a loading control (**Q**). Densitometric analysis of nSMase2 expression (**R**). All experiments were performed in triplicates. (**S and T**) Representative Western blot showing CD63 expression in mock, JEV-infected cells (18 hpi), and poly(I:C)-transfected cells (18 h post-transfection) (**S**). β-actin served as a loading control. (**T**) Densitometric quantification of CD63 normalized to β-actin. Experiments were done in triplicates. (**U**) Flow cytometry analysis of %MFI relative to mock in JEV-infected and poly(I:C)-transfected Neuro2a cells at indicated time points. Experiments were done in triplicates. (**V**) NTA of EVs from supernatants of JEV-infected (18 hpi) and Poly I:C-transfected (18 h post-transfection) cells. Experiments were done in triplicates. Statistical analysis was performed using one-way ANOVA with multiple comparisons. *P < 0.05, **P  <  0.01, ***P  <  0.001, ****P  <  0.0001; ns,not significant.

LDs, which originate from the ER, store neutral lipids and are increasingly recognized as key organelles in viral replication and assembly (42). Prior studies have demonstrated the involvement of LDs in JEV replication (24). In our study, immunostaining microscopy (Fig. 5A through C) and FACS (Fig. 5D) analysis revealed a dynamic modulation of LDs during JEV infection in Neuro2a cells. Specifically, both the number and size of LDs increased from 2 to 6 h post-inoculation (hpi), followed by a progressive decline through 24 hpi (Fig. 5A through D; Fig. S4A). Similarly, immunohistochemical analysis demonstrated increased LD biogenesis in JEV E protein–positive cells in the hippocampal region of brain tissues inoculated with either form of JEV—enclosed within vesicles and as free virus. Hence, validating increase in LD biogenesis with JEV infection is physiologically relevant. Further, the percent of BODIPY^+^ JEV^+^ cells was significantly higher in JEV vesicle–inoculated brain tissues compared to free JEV infection, correlating with infectivity (Fig. S5A and B; Fig. 3I). Interestingly, the reduction in intracellular LD content beginning at 14 hpi coincided with an increase in sEV release, as quantified by NTA (Fig. 5E). This inverse correlation suggested a potential role for LDs in sEV-mediated viral egress.

To further demonstrate the functional role of LDs in this context, we inhibited LD biogenesis using inhibitors targeting DGAT1 and DGAT2, enzymes that convert diacylglycerol (DAG) to triacylglycerol (TAG), the core component of LDs. Short-term treatments (2–6 h) were optimized to minimize cytotoxicity and prevent disruption of JEV replication (Fig. 5F). Western blotting of treated cells showed decreased expression of Plin2, a structural LD membrane protein, confirming LD depletion (Fig. 5F and G). However, JEV capsid expression did not reduce following 2 h of inhibition of DGAT1 + 2, validating intact replication (Fig. 5F and I). Notably, nSMase2 levels also declined under these conditions (Fig. 5F and H), along with viral titers in isolated sEVs (Fig. 5J). NTA showed that DGAT1 + 2 inhibition for 2, 4, and 6 h reduced extracellular vesicle release in JEV-infected Neuro2a cells (Fig. 5K), suggesting a mechanistic link between LD abundance, ceramide-mediated sorting, and viral release.

Further, we questioned whether the reduction in JEV egress observed in Fig. 5J is due to reduced virus release through the secretory pathway or specifically to reduced sEV biogenesis. To answer this, supernatants after treatment with DGAT1 + 2 were incubated with anti–JEV E protein to neutralize free JEV particles and quantify the amount of JEV still present in the sample. Viral genome levels showed significant reduction in quantification post-neutralization with anti-JEV E protein (Fig. 5L). This suggests that, post DGAT1 + 2 treatment, the JEV released in supernatant was contributed by sEV-free JEV particles that were neutralized by anti-JEV E protein. Hence, LD biogenesis plays a significant role in EV-mediated JEV egress.

We examined the role of lipid droplet (LD) biogenesis in EV-mediated JEV egress by knocking down Seipin, a key ER-resident protein regulating LD shape, formation, and maturation (43, 44), using 50 μM siRNA-mediated knockdown in Neuro2a cells. Loss of Seipin resulted in reduced CD63 (multivesicular body [MVB], marker)–LD colocalization in JEV-infected cells, indicating impaired MVB-LD interaction (Fig. S6A and B). Western blot analysis confirmed efficient silencing of Seipin, accompanied by reduced Alix, CD63, Plin2, and JEV capsid expression (Fig. 5M; Fig. S6C through G). Consistent with a role for LDs in viral replication, assembly, and egress, NTA demonstrated a reduction in sEV release following Seipin knockdown (Fig. 5N). Together, these findings indicate that disruption of Seipin-dependent LD biogenesis compromises JEV replication, MVB formation, and release of JEV by sEVs.

Immunostaining of Neuro2a cells at 18 hpi showed colocalization of BODIPY-labeled LDs and nSMase2, which was enhanced upon oleic acid (OA) treatment—known to promote LD biogenesis—and diminished upon DGAT1 + 2 inhibition (Fig. 5O and P; Fig. S4B and C). This further supports the interplay between LD generation and ceramide pathway activation during JEV infection. To directly examine nSMase2 association with LDs, we isolated LD fractions from Neuro2a cells infected with JEV (18 hpi), with or without OA treatment. Immunoblotting revealed a significant enrichment of nSMase2 in the LD fraction upon OA stimulation, relative to Plin2 levels (Fig. 5Q and R), indicating active recruitment of nSMase2 to LDs during infection.

To determine whether LD remodeling and enhanced sEV release are specific to JEV infection or represent a general response to double-stranded RNA (dsRNA), which forms an intermediate in most replicating RNA viruses, Neuro2a cells were transfected with Poly I:C for 24 h, a synthetic dsRNA mimic. Plin2 expression was observed to increase at different concentrations of Poly I:C (Fig. S6H and I). Poly I:C stimulation increased expression of the MVB marker CD63 (Fig. 5S and T), LD secretion (Fig. 5U), and sEV release compared to mock-treated cells (Fig. 5V). These findings indicate that LD biogenesis and sEV upregulation represent a general cellular response to dsRNA stimulation rather than a phenomenon restricted to JEV infection. Western blot analysis further revealed increased levels of nSMase2 and ceramide kinase upon Poly I:C treatment compared to JEV-infected conditions (Fig. S6 through M). The upregulation of these enzymes indicates activation of the ceramide-dependent sEV biogenesis pathway. Collectively, these findings demonstrate that dsRNA stimulation is sufficient to trigger LD biogenesis and sEV secretion through ceramide-dependent pathway activation.

**Fig 6:**
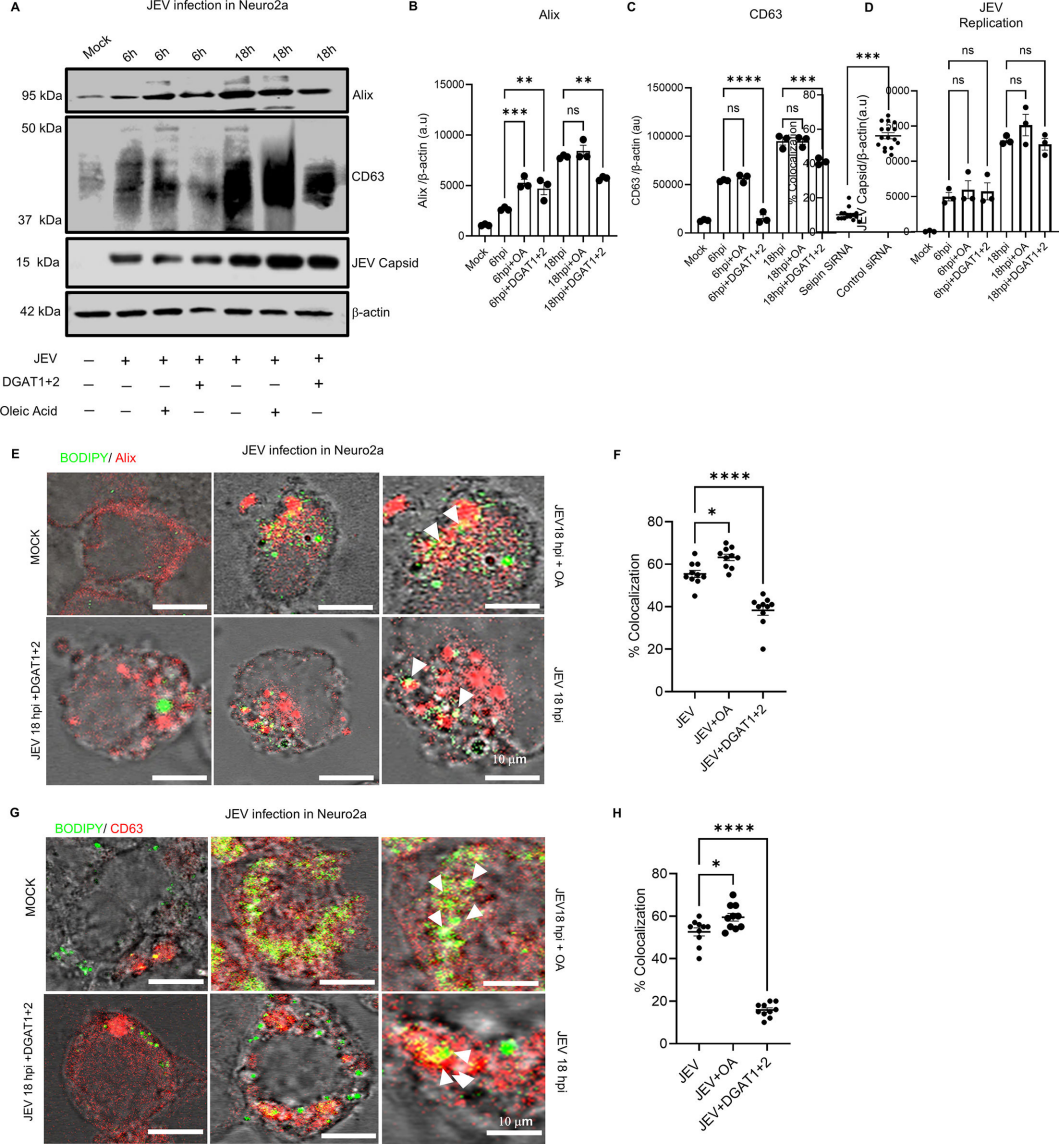
Lipid droplet (LD)–MVB interactions regulate sEV-mediated JEV egress. (**A**) Representative Western blot showing Alix, CD63, and JEV capsid protein expression in Mock and JEV-infected Neuro2a cells at 6 and 18 hpi under the following conditions: JEV 6 hpi, JEV 6 hpi + OA, JEV 6 hpi + DGAT1 + 2 inhibitors, JEV 18 hpi, JEV 18 hpi + OA, and JEV 18 hpi + DGAT1 + 2 inhibitors (**A**). β-actin was used as a loading control. (**B–D**) Densitometric quantification of (**B**) Alix, (**C**) CD63, and (**D**) JEV capsid levels normalized to β-actin. Experiments were done in triplicates. (**E**) Representative immunostaining images showing colocalization of LD with Alix in mock, JEV 18 hpi, JEV 18 hpi + OA, and JEV 18 hpi + DGAT1 + 2 inhibitors (2 h)–treated Neuro2a cells. Scale bar = 10 µm. Arrowheads indicate regions of LD–Alix colocalization. Representative images from one out of three biologically independent experiments were shown. (**F**) The percentage of colocalization relative to the total number of cells in each field was quantified. Each data point represents the percentage of LD–Alix colocalization per field. (**G**) Representative immunostaining images showing colocalization of LD with CD63 in mock, JEV 18 hpi, JEV 18 hpi + OA, and JEV 18 hpi + DGAT1 + 2 inhibitors (2 h)–treated Neuro2a cells. Scale bar = 10 µm. Arrowheads indicate regions of LD–CD63 colocalization. Images are representative of three biologically independent experiments. (**H**) The percentage of colocalization relative to the total number of cells in each field was quantified. Each data point represents the percentage of LD–CD63 colocalization per field. Statistical analysis was performed using one-way ANOVA with multiple comparisons. *P < 0.05, **P  <  0.01, ***P  <  0.001, ****P  <  0.0001; ns, not significant.

A recent study by Grenard et al. (27) reported increased CD63^+^ and Alix^+^ MVBs in irradiated pancreatic cancer cells, accompanied by elevated LD accumulation, suggesting a potential role for LDs in MVB biogenesis. We examined the expression of the MVB markers Alix and CD63 during JEV infection in Neuro2a cells under conditions that modulate LD abundance. Western blot analysis revealed that Alix and CD63 protein levels increased at 6 and 18 h post-infection (hpi) compared with mock-treated controls, and this increase was further enhanced by OA-induced LD biogenesis and attenuated following DGAT1 + 2 inhibition (Fig. 6A through D). Consistently, confocal microscopy showed that DGAT1 + 2 inhibitor treatment significantly reduced Alix and CD63 signals at both 6 and 18 hpi and decreased their colocalization with LDs, whereas OA treatment enhanced their colocalization with LDs (Fig. 6E and H; Fig. S7A through C). These observations suggest that LDs spatially associate with MVBs and may supply lipid components required for MVB maturation and sEV formation.

Western blot analysis revealed that OA treatment increased the expression of Alix and CD63, whereas inhibition of LD formation using a DGAT inhibitor reduced their levels in the absence of JEV infection (Fig. S6N throughQ). Consistently, OA treatment enhanced sEV release under these conditions, indicating that LD induction alone is sufficient to promote sEV secretion (Fig. S6R). Collectively, our results indicate that JEV exploits host LD machinery to promote ceramide-dependent, ESCRT-independent sEV secretion, tightly coupled to viral loading and release. However, at this stage we do not know the molecular mechanism of how JEV virions are packaged inside MVBs, which needs further investigation.

### nSMase2 regulates the crosstalk between lipid droplet biogenesis and JEV egress via small extracellular vesicles

**Fig 7:**
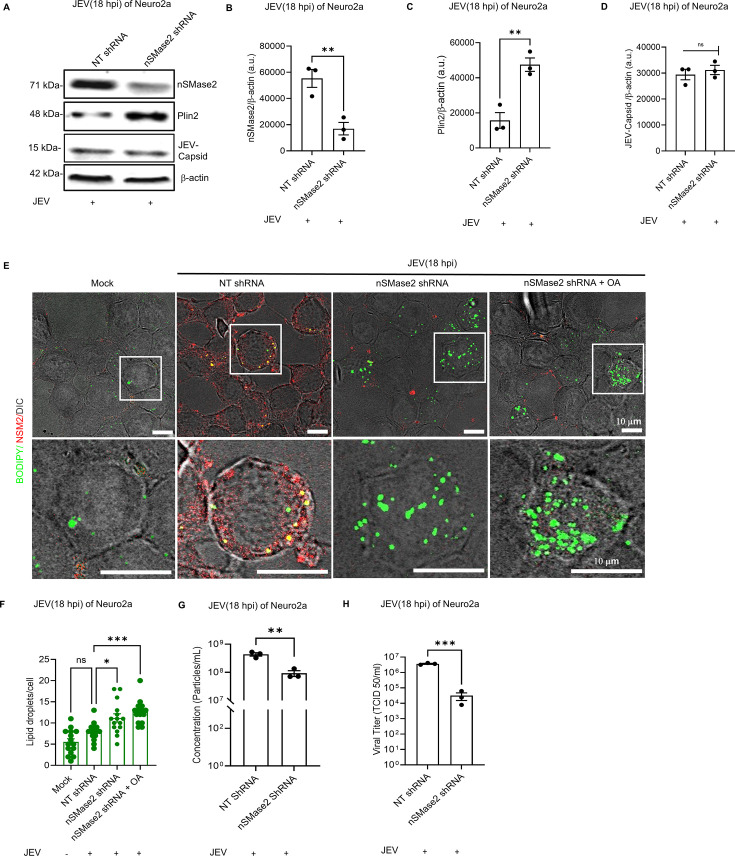
nSMase2 knockdown inhibits JEV release via sEVs and enhances lipid droplet (LD) secretion. (**A**) Representative Western blot showing the expression levels of nSMase2, Plin2, and JEV capsid protein in Neuro2a cells transfected with non-targeting (NT) shRNA or nSMase2 shRNA, followed by JEV infection (18 hpi). β-actin was used as the loading control. (B–D) Densitometric quantification of (**B**) nSMase2, (**C**) Plin2, and (**D**) JEV capsid protein, normalized to β-actin. Experiments were done in triplicates. (**E**) Representative immunostaining images showing distribution of LD and nSMase2 under the following conditions: mock, JEV (18 hpi) + NT shRNA, JEV (18 hpi) + nSMase2 shRNA, and JEV (18 hpi) + nSMase2 shRNA + OA. Scale bar = 10  µm. Images are representative of three biologically independent experiments. Lower panels show magnified views of the white boxed regions. (**F**) Quantification of LDs per cell corresponding to the images in panel E. Each data point represents an individual cell (*n* = 15 per group) in a field. (**G**) Quantification of vesicle concentration (particles/mL) in supernatants from JEV-infected cells treated with NT shRNA or nSMase2 shRNA. Experiments were done in triplicates. (**H**) JEV titer (TCID50/mL) measured from sEVs isolated from JEV-infected cells treated with NT shRNA or nSMase2 shRNA. Experiments were done in triplicates. Statistical analysis was performed using one-way ANOVA with multiple comparisons and unpaired *t*-tests for pairwise comparisons. Data represent three independent experiments *P < 0.05, **P  <  0.01, ***P  <  0.001; ns, not significant.

To further elucidate the role of nSMase2 in linking LD dynamics with JEV release via sEVs, both knockdown and overexpression experiments were performed in Neuro2a cells. Silencing of nSMase2 using specific shRNA resulted in a significant reduction in nSMase2 protein levels compared to non-targeting (NT) controls, accompanied by a notable increase in Plin2 expression, indicating intracellular LD accumulation. However, no significant change in JEV capsid protein expression was observed (Fig. 7A through D). These findings suggest that while nSMase2 does not play a role in viral replication, it is critical in mediating JEV egress. Immunostaining revealed increased LD abundance and a more cytoplasmic localization pattern in nSMase2-depleted cells, as visualized by BODIPY staining (Fig. 7E and F). Notably, this was observed both with and without OA treatment. In contrast, NT control cells infected with JEV displayed more peripheral colocalization of LDs with nSMase2 (as seen in Fig. 5O, P, 7E and F; Fig. S8A and B). NTA further revealed a significant decrease in sEVs concentration in the nSMase2 knockdown group (Fig. 7G), and viral titers recovered from these vesicles were substantially reduced (Fig. 7H), reinforcing the role of nSMase2 in sEV-mediated viral export.

**Fig 8:**
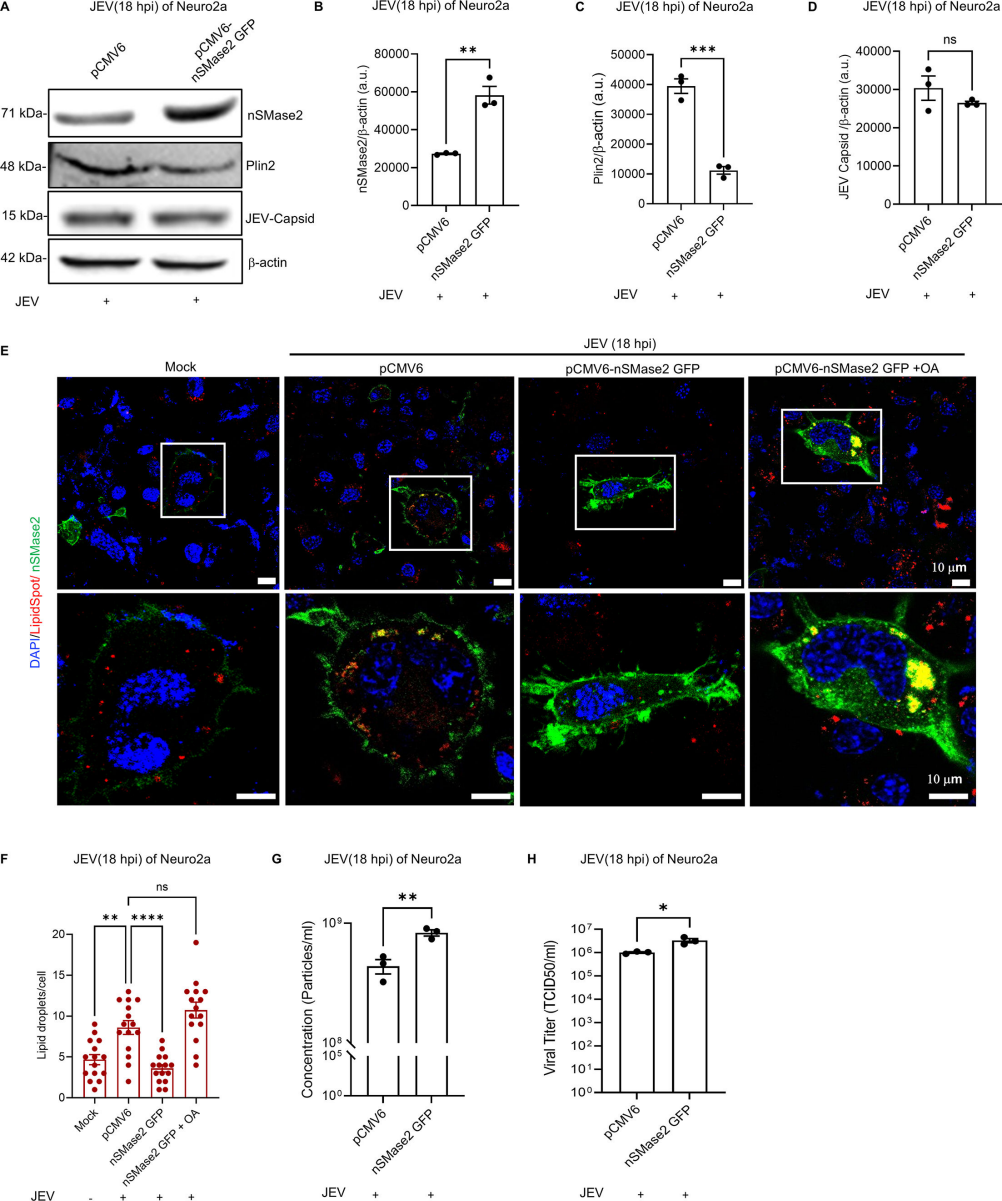
Overexpression of nSMase2 suppresses LD secretion during JEV infection in Neuro2a cells. (**A**) Representative Western blot showing protein levels of nSMase2, Plin2, and JEV capsid in Neuro2a cells transfected with either control vector (pCMV6) or nSMase2-GFP construct (pCMV6-nSMase2-GFP), followed by JEV inoculation (18 hpi). β-actin served as the loading control. (**B–D**) Quantitative densitometry of (**B**) nSMase2, (**C**) Plin2, and (**D**) JEV capsid levels normalized to β-actin. Data represent three independent experiments (**E**) Representative Immunostaining images illustrating the distribution of LD (LipidSpot staining) and nSMase2 under various conditions: mock, JEV (18 hpi) + pCMV6, JEV (18 hpi) + nSMase2 GFP, and JEV (18 hpi) + nSMase2 GFP + OA. Scale bar = 10 µm. Lower panels show magnified views of the white boxed regions. Images are representative of three biologically independent experiments. (**F**) Quantification of the number of lipid droplets per cell across the experimental groups. Each data point represents an individual cell (*n* = 15 per group). (**G**) Quantification of vesicle concentration (particles/mL) in supernatants from JEV-infected cells treated with control vector (pCMV6) or nSMase2-GFP construct (pCMV6-nSMase2-GFP). Experiments were done in triplicates. (**H**) JEV titer (TCID50/mL) measured from sEVs isolated from JEV-infected cells treated with control vector (pCMV6) or nSMase2-GFP construct (pCMV6-nSMase2-GFP). Experiments were done in triplicates. Statistical analysis was performed using one-way ANOVA followed by multiple comparisons or unpaired *t*-tests, as appropriate. *P  <  0.05, **P  <  0.01, ***P  <  0.001, ****P  <  0.0001; ns, not significant.

In contrast, overexpression of nSMase2 through pCMV6-nSMase2-GFP transfection led to elevated nSMase2 levels and a concurrent reduction in Plin2 expression, without affecting JEV capsid levels (Fig. 8A through D). This phenotype suggests that the enhanced turnover or utilization of LDs occurs in the presence of excess nSMase2. Immunostaining showed reduced LD accumulation, increased membrane, and ERGIC-associated nSMase2 in JEV-infected cells (Fig. 8E and F; Fig. S8C through F). Consistent with these findings, sEVs isolated from nSMase2-overexpressing cells displayed a significant increase in vesicle concentration (Fig. 8G) and JEV titers (Fig. 8H), confirming enhanced vesicular export of infectious particles. Together, these gain and loss-of-function studies strongly support that nSMase2 acts as a central regulator of the ceramide-mediated, ESCRT-independent sEV biogenesis pathway exploited by JEV. Through modulating LD availability and localization, nSMase2 facilitates the incorporation of viral cargo into sEVs, thereby promoting efficient, non-lytic viral egress.

## DISCUSSION

Lipid droplets (LDs) and extracellular vesicles (EVs), which are distinct cellular components, have recently been shown to have overlapping functional landscapes. Traditionally regarded as separate—LDs serving as lipid storage depots and EVs as mediators of intercellular communication—both have now been implicated in key cellular and pathogenic processes (27, 45). Emerging studies indicate that LDs and EVs play a crucial role in viral replication, intracellular trafficking, virion assembly, and unconventional secretion mechanisms (24, 42, 46, 47). A significant conceptual advance emerged from the realization that LDs are not merely for lipid homeostasis and lipid storage sites, but also actively engage in membrane trafficking and organelle crosstalk (48, 49). This includes interactions with endolysosomal compartments and multivesicular bodies (MVBs), the precursors of small EVs (sEVs), as shown by Genard et al. (27), who provided the first direct experimental link between LD biogenesis and sEV release mediated through RAB5C. In the context of viral infections, while both enhanced LD formation and sEV-mediated viral release have been observed independently, the molecular connection between these organelles during viral infection remains poorly defined. Our study addresses this gap by dissecting the mechanistic interplay between LDs and sEVs in JEV-infected neuronal cells and Poly I:C-transfected neuronal cells (3, 50–52).

Xiong et al. (13) in their recent report showed that JEV can be released non-lytically within sEVs from PIEC, HeLa, and BHK-21 cells (13). In line with their findings, our study provides additional evidence that JEV is similarly released non-lytically from neuronal cells, including Neuro2a, SHSY-5Y (a neuronal lineage), N9 (microglial cells), and primary cortical neurons, within vesicles ~200 nm in diameter. Compared to JEV released through the secretory pathway, the JEV population packaged inside sEVs has a higher proportion of premature membrane protein (PrM) compared to membrane protein, as indicated by a higher ratio of PrM/M. Vesicle-cloaked JEVs were observed to be more infectious than the vesicle-free virions both *in vitro* and *in vivo* models. Collectively, these findings suggest that the sEV egress may serve as an adaptive mechanism, facilitating the egress of a higher proportion of immature JEV particles and thereby conferring enhanced infectivity advantages. This process may enable a more rapid mode of viral transmission through vesicle-mediated mechanisms evading host antibodies.

Our data show that JEV explicitly uses the ESCRT-independent pathway mediated by neutral sphingomyelinase 2 (nSMase2) and the ceramide biosynthesis pathway for its egress inside sEVs. The role of nSMase2 in sEV biogenesis is well characterized in neurons, particularly in the release of tau protein and factors that promote neuronal survival and neuroprotection (17, 53, 54). Our findings reveal that JEV hijacks this endogenous machinery for its release via secreted extracellular vesicles (sEVs). Notably, although Xiong et al. reported that GW4869—a selective inhibitor of nSMase2—disrupted JEV replication and sEV release when used in pre-treatment, our study adopted a more targeted approach. We pre-treated infected cells with 10 µM and 20 µM of GW4869 for a minimum of 12 h, ensuring that cytotoxicity was avoided. Under these conditions, JEV replication remained unaltered, but its release in sEVs was substantially inhibited, underscoring the role of the ceramide-nSMase2 axis specifically in viral packaging and egress. The role of LDs in JEV replication and assembly has been previously reported (24, 55). We also observed a sharp increase in LD accumulation up to 6 h post-infection (hpi), followed by a decline from 14 h post-infection (hpi) onward. Interestingly, this temporal pattern inversely correlated with a marked increase in sEV release beginning around 14 hpi. These sequential dynamics prompted us to explore a potential functional linkage between LD dynamics and sEV-mediated viral release in JEV infection. This novel aspect may also extend to other viral systems.

It was a compelling observation that the reduction in LD number and size during JEV infection could be attributed either to a halt in LD biogenesis or to their functional consumption—(i) in supporting viral replication or (ii) in mediating sEV egress, or possibly both. Given the critical role of LDs in JEV replication, as highlighted in earlier studies, we adopted a cautious approach when inhibiting LD synthesis to ensure that viral replication remained unperturbed. Indeed, a short 2-hour treatment with dual DGAT1 and DGAT2 inhibitors—known to block LD biogenesis—did not affect JEV replication, yet significantly hampered the release of JEV within sEVs. Interestingly, at 18 h post-infection (hpi), we observed that LDs were positioned peripherally and appeared to colocalize with nSMase2. This was further substantiated by our subcellular LD fractionation experiments, which revealed enrichment of nSMase2 in the LD fraction. In the context of metabolic disorders such as non-alcoholic fatty liver disease (NAFLD), often associated with type 2 diabetes and impaired immunity, LD accumulation is reported to correlate with ceramide (Cer) generation and nSMase2 expression on LD membranes (29). However, in our model, we specifically detected this colocalization only at 18 h post-infection (hpi). Since nSMase2 is known to traffic intracellularly between the Golgi and plasma membrane (56), it may encounter LDs transiently during endosome maturation and multivesicular body (MVB) formation. LDs have previously been implicated in providing membrane lipids for MVB biogenesis, and given that nSMase2 is also crucial for intraluminal vesicle formation within MVBs, it is plausible that the observed colocalization at 18 hpi represents a functional convergence during sEV biogenesis. Direct physical or functional interactions between LDs and nSMase2 remain poorly understood. Our study, therefore, aimed to elucidate this interface in the context of JEV infection.

To dissect the functional relevance of nSMase2 in JEV trafficking, we employed shRNA-mediated knockdown of nSMase2 in infected cells. This resulted in a significant increase in the number of LDs, as evidenced by enhanced expression of LD-resident membrane protein Plin2. This increase in LD biogenesis suggests a disruption in LD turnover and utilization. In control (non-target/scrambled) conditions at 18 hpi, LDs exhibited peripheral localization and showed spatial colocalization with nSMase2, possibly implicating their localization on the endosomal compartment (27, 41). However, in nSMase2-depleted cells, LDs were redistributed across the cytoplasm and cell periphery without colocalization with nSMase2. Since nSMase2 catalyzes the hydrolysis of sphingomyelin into ceramide, it plays a pivotal role in cellular processes. It is involved in the budding of intraluminal vesicles (ILVs) within multivesicular bodies (MVBs). Additionally, it contributes to small extracellular vesicle (sEV) biogenesis and cargo loading; its knockdown led to a substantial reduction in sEV secretion, as expected (16, 17, 57). Notably, this reduction in sEV release was paralleled by diminished release of JEV, suggesting that nSMase2-driven ceramide enrichment is necessary for JEV export through the sEV pathway. This finding aligns with previous observations, where ceramide-mediated membrane curvature facilitates ILV formation—a prerequisite for exosome generation (58–60).

The concurrent increase in LD numbers in nSMase2-deficient cells likely reflects a compensatory metabolic bottleneck. Under normal infection conditions, LDs may provide structural lipids, support membrane curvature, or serve as organelle contact surfaces that facilitate MVB maturation or cargo sorting. In the absence of efficient sEV formation, this downstream demand is attenuated, leading to an intracellular accumulation of LDs. Thus, our data suggest that LDs, although transiently associated with the egress process, are not directly involved in transporting JEV virions to the plasma membrane; instead, they serve in the assembly of MVBs. This conclusion is further substantiated by the inverse phenotype upon nSMase2 overexpression: a marked decrease in Plin2 levels and LD abundance, accompanied by enhanced sEV and viral release. We interpret this as LDs being actively consumed during MVB formation and viral packaging when the sEV biogenesis pathway is fully operational. It is also likely that the ceramide-enriched domains facilitated by nSMase2 may interact with LDs at membrane contact sites (MCSs), promoting lipid flux or localized membrane remodeling that drives MVB biogenesis.

In summary, our study investigates the poorly understood coordination between LDs and nSMase2 in sEV biogenesis and JEV egress. We found that LDs are not directly involved in viral egress but contribute to multivesicular body (MVB) formation. LDs and nSMase2 have independent yet synchronized roles in facilitating JEV trafficking and release. However, our reliance on *in vitro* models limits the understanding of LD-nSMase2 dynamics *in vivo*, especially within the brain’s complex environment. High-resolution and spatial transcriptomics, combined with organelle-specific analyses, could clarify the sequence of events during viral egress. Despite these limitations, our findings have translational relevance. The study suggests that organelle interactions are potential therapeutic targets, and studying EV formation and cargo could aid in the development of biomarkers for disease onset and severity. Future research using patient samples is crucial for translating these insights into detection and treatment strategies for JEV outbreaks.

## MATERIALS AND METHODS

### Cell culture and treatments

The murine neuroblastoma cell line Neuro2a and the microglial cell line N9 were provided as gifts by Dr. Anirban Basu, National Brain Research Centre (NBRC), India, and Dr. Prem Tripathi, CSIR-Indian Institute of Chemical Biology (IICB), India, respectively. The human neuroblastoma cell line SHSY-5Y (Ref no. 811/2024-25) was sourced from the National Centre for Cell Science (NCCS), Pune, India. The mouse neuroblastoma cell line Neuro2a, human neuroblastoma cell line SHSY-5Y, and mouse microglial cell line N9 were cultured in 1× DMEM (Gibco, USA) supplemented with 10% fetal bovine serum (FBS; Gibco, USA) and 1× antibiotic-antimycotic solution (Gibco, USA). Cells were maintained at 37°C in a humidified incubator with 5% CO₂.

Murine primary cortical neurons were cultured as previously described with modifications (61). Briefly, neocortices were isolated from embryonic day 16–18 (E16–18) mouse embryos and dissected in serum-free DMEM/F12 medium (Gibco, USA) (1:1), supplemented with 6 mg/mL D-glucose (SRL, India), 100 µg/mL apo-transferrin (Sigma-Aldrich, Germany), 25 µg/mL insulin (Sigma-Aldrich, Germany), 20 nM progesterone (Sigma-Aldrich, Germany), 60 µM putrescine (Sigma-Aldrich, Germany), and 30 nM selenium (Sigma-Aldrich, Germany), with penicillin-streptomycin (Thermo Scientific, USA). The tissue was gently triturated to obtain a single-cell suspension, which was then centrifuged for 5 min at 1,000 rpm at room temperature. The cells were resuspended in cortical medium and seeded at 1.5 million per well of a 6-well plate onto poly-D-lysine (Sigma-Aldrich, Germany)-coated plates. One day after *in vitro* incubation, the cells were maintained in neurobasal medium (Thermo Scientific, USA). The neurons were inoculated or treated after 5 days of plating.

Neuro2a cells infected with JEV were treated with Brefeldin A (BFA; 5 µg/mL; Sigma-Aldrich) at 20–2 h post-infection (hpi). Cells and supernatants were collected at 24 hpi (4–24 h of BFA exposure). Cell supernatants were used for extracellular viral RNA and Gaussia luciferase assays, while corresponding cell lysates were analyzed to assess intracellular viral replication.

For GW4869 (MCE, USA) treatment, Neuro2a cells were seeded in 6-well plates at a density of 0.3 × 10^6^/well 24 h post-seeding, the medium was replaced with fresh incomplete medium, and JEV was inoculated (MOI 5). After 2 h, the inoculum was removed, and fresh medium was added along with different concentrations of GW4869 (10 µM, 20 µM) at the indicated time points, and cell lysates and supernatants were collected for subsequent experiments.

For oleic acid (Sigma-Aldrich, Germany) treatment, the cells were seeded and incubated with 30 µM of oleic acid (OA) overnight. The next day, OA-containing medium was removed and replaced with fresh complete medium.

For DGAT1 + 2 (Cayman, USA) treatment, cells were seeded, and the DGAT1 + 2 inhibitor was added post-inoculation at a concentration of 50 µM. At different time points, samples were collected for subsequent experiments.

### Gaussia luciferase assay

Gaussia luciferase activity was measured using the Pierce Gaussia Luciferase Glow Assay Kit (Thermo Scientific, USA). Neuro2a cells were electroporated with a Gaussia luciferase expression plasmid (pCMV-Gaussia-Dura Luc Vector, Thermo Scientific) and subjected to mock or JEV infection, with or without brefeldin A (BFA) treatment. Culture supernatants were collected and assayed for Gaussia luciferase activity according to the manufacturer’s instructions. Briefly, 15 µL of supernatant was mixed with 50 µL of working solution in a black 96-well plate, and luminescence was measured after 10 min using a SpectraMax M5 Multi-Mode Microplate Reader.

### Virus infection

The JEV strain GP78 was a gift from Dr. Anirban Basu, National Brain Research Centre (NBRC), India. All experiments related to JEV infection in this work are approved by the Institutional Bio-safety Committee (IBSC) of CSIR-IICB (No. CSIR-IICB/IBSC/CERT-87/24). For virus infection, cells were seeded 24 h prior to inoculation. Upon reaching 80–90% confluency, the cells were washed twice with 1× PBS and then incubated with incomplete medium. Subsequently, the cells were inoculated with virus at a multiplicity of infection (MOI) of 5 from the viral stock (10^8^ TCID50/mL) and incubated at 37°C in a 5% CO₂ incubator for 2 h. After incubation, the inoculum was removed, and the cells were washed twice with incomplete DMEM. Fresh incomplete medium was then added for further incubation.

### Viral stock preparation and TCID50 assay

A virus dilution was prepared from a laboratory stock of 1 × 10⁸ pfu/mL. Postnatal day-3 (P-3) mouse pups were injected intracranially with diluted virus suspension for virus stock preparation. After 3–4 days post-infection, when symptoms were prominent, brains were harvested. For virus extraction, each brain was subjected to sonication using 30-second pulses with 5-minute intervals, repeated for a total of three times. The homogenized samples were then centrifuged at 15,000 rpm for 45 min at 4°C. The supernatant was carefully collected and filtered through a 0.22 µm filter. Viral aliquots were stored at –80°C until further use.

For titration, Neuro2a cells were seeded in 96-well plates at a density of 0.1 × 10⁵ cells per well 24 h before inoculation. Serial dilutions of the virus stock or extracellular medium were prepared in incomplete DMEM (Gibco, USA). The diluted virus was added to the cells in quadruplets, and cytopathic effects (CPE) were monitored daily for up to 5 days post-inoculation. After the incubation period, the medium was discarded, and 100 µL of crystal violet solution (HIMEDIA, India) was added (0.25% crystal violet supplemented with 20% ethanol in double-distilled water). The solution was then incubated for 15 min. The solution was removed, wells were gently washed twice with distilled water, and an image of the stained 96-well plates was captured for analysis. The dilution that showed 50% cell death—that is, approximately 50% of the well stained with crystal violet, indicating surviving cells—was used to calculate the TCID50/mL, following the method described in reference 62. The virus stock we used for our experiments had a titration value of 1 × 10^8^ TCID50/mL, and the limit of detection (LOD) for JEV was determined to be 1 × 10^2^ TCID50/mL.

### Standard curve preparation for quantitative PCR

Double-stranded DNA (dsDNA) amplicons corresponding to each primer set were synthesized by Barcode Biosciences. The number of amplicon copies was calculated from the measured DNA mass and amplicon length using the equation Number of copies (molecules) = *X* (ng) × 6.0221 × 10²³ / (*N*/2) × 660 g·mol⁻¹) × 10⁹ (ng·g⁻¹), where *X* is the amount of amplicons in nanograms, *N* is the length of the dsDNA amplicon, 660 g mol^−1^ is the average mass of 1 bp dsDNA, and 6.0221 × 10^23^ is Avogadro’s number.

Each synthesized amplicon was resuspended in 1 mL of DNase/RNase-free water to obtain the initial stock concentration expressed as copies per milliliter. From this stock, tenfold serial dilutions ranging from 10¹⁰ to 10¹ copies per milliliter were prepared and used to generate standard curves for each primer set.

Quantitative PCR (qPCR) was performed using 10 µL reaction volumes. Standard curves were generated by plotting threshold cycle (Ct) values against the log₁₀-transformed amplicon copy numbers per milliliter. Linear regression analysis was used to obtain the standard curve equation and correlation coefficient (*R*²). These curves were subsequently used to calculate viral genome copy numbers in unknown samples. We used the standard curve equation *y* = −2.2259*x* + 36.061, where *y* = Ct value and *x* = log₁₀ RNA copies/mL.

### Trypan blue staining for plasma membrane permeability/cell viability

Cells were seeded at a density of 0.3 × 10^6^ cells/well in 6-well plates and exposed to different treatments. At various time points after infection, cells were collected from each treatment group. After trypsinization with 0.25% Trypsin-EDTA (Gibco, USA), the cells were centrifuged at 3,000 rpm for 5 min, and the pellet was resuspended in 1 mL of complete DMEM containing 10% FBS. A 1:1 mixture of the cell suspension and 0.4% trypan blue stain (Thermo Scientific, USA) was prepared and incubated at room temperature for 3 min. Then, 10 µL of the mixture was loaded onto a hemocytometer chamber slide to assess cell viability. The cell number was calculated by an automated cell counter (Invitrogen, USA).

### Isolation and purification of sEV

For EV isolation, cells were seeded in 100 mm tissue culture dishes 24 h before JEV inoculation. Cultures at 80–90% confluency were washed with incomplete medium and switched to FBS-free medium, followed by infection with JEV at a MOI of 5. Culture supernatants were collected between 18 and 34 hpi and sequentially centrifuged at 300 × *g* for 10 min to remove intact cells, 2,000 × *g* for 10 min to eliminate dead cells, and 10,000 × *g* for 30 min to deplete large vesicles and cellular debris. The supernatant was then ultracentrifuged at 100,000 *× g* for 2 h at 4°C (fixed-angle rotor, Beckman Coulter), and the resulting small EV-enriched pellet was gently resuspended in sterile PBS for density-gradient purification. Iodixanol gradients were prepared with four layers arranged from top to bottom as 5%, 10%, 20%, and 40% iodixanol. Iodixanol gradients were prepared by first generating a 40% iodixanol solution from 60% OptiPrep (Abcam, UK) diluted in working solution buffer (0.25 M sucrose, 6 mM EDTA, 60 mM Tris-HCl, pH 7.4). Subsequently, 20%, 10%, and 5% iodixanol layers were prepared by diluting the 40% stock in homogenization buffer (0.25 M sucrose, 1 mM EDTA, 10 mM Tris-HCl, pH 7.4). The EV suspension was overlaid onto the 5% layer and centrifuged at 100,000 × *g* for 16 h in a swinging-bucket rotor (SW-41 Ti rotor, Beckman Coulter, USA). Three distinct interfaces were collected: interface 1 (I1) between 5% and 10% iodixanol layers, interface 2 (I2; EV-enriched) between the 10% and 20% iodixanol layers, and interface 3 (I3; vesicle-free virus–enriched) above the 40% iodixanol layer. Interfaces were washed in PBS, followed by a second ultracentrifugation at 100,000 × *g* for 90 min to remove residual iodixanol. All EV isolation steps were performed at 4°C.

The EVs from the I2 interface were further subjected to CD63-positive selection using Protein A/G magnetic beads (MedChemExpress, USA) according to the manufacturer’s instructions. Briefly, the sEV suspension was incubated with anti-CD63–coated beads for 2 h at 4°C with gentle rotation, washed three times with PBS, and eluted for downstream applications.

### Generation of intact JEV vesicle and free JEV preparations

Intact JEV-containing sEVs were isolated from supernatants derived from Neuro2a cells infected with JEV at a MOI of 5 and harvested at 18 hpi by iodixanol density-gradient ultracentrifugation at 100,000 × *g*. The obtained sEVs were divided into two equal fractions, where one fraction remained intact and was diluted in 1× DPBS, and the second fraction was treated with hypotonic lysis buffer (1:1, bioWORLD, USA) and incubated overnight at 4°C to yield free-virus preparations. A volume-matched control was prepared by adding equal amounts of PBS to the intact sEV fraction. Both preparations were used either for intracranial inoculation of 3–4-day-old BALB/c pups or infection of Neuro2a cells. Finally, viral infectivity was quantified by TCID50 assay, and RNA copy numbers were determined by qRT-PCR.

### Mouse experiments

To determine the pathogenicity of EVs and EV-free virions, mice were injected intracranially with 25 µL of either EVs or EV-free virions, using equal volume based on RNA copy numbers. Mice in the control group were injected with EVs derived from mock-infected cells. Body weight, clinical symptoms, and survival rates were monitored daily. At 5 or 7 dpi, mice from each group were sacrificed, and the brain tissues were collected. The viral loads and mRNA levels of indicated cytokines in the brain tissues were determined by TCID50/mL and qRT-PCR, respectively.

Three-day-old BALB/c mouse pups were divided into three groups (*n* = 5 per group): mock (PBS), JEV vesicle, and Free JEV. Mice were intracranially inoculated with equivalent volumes of the respective preparations and monitored daily for survival and body weight. Survival was recorded for up to 8 days post-inoculation. Body weight was measured daily and expressed as a percentage of initial weight. All experiments were performed in triplicate.

### Isolation and purification of multivesicular body (MVB)

Multivesicular bodies (MVBs) were isolated from cultured cells using a differential centrifugation method followed by sucrose density gradient ultracentrifugation. Briefly, cells were washed with ice-cold phosphate-buffered saline (PBS) and harvested by scraping in PBS supplemented with protease inhibitors (Sigma-Aldrich, Germany). The cell pellets were resuspended in homogenization buffer containing 250 mM sucrose (SRL, India), 1 mM EDTA (SRL, India), and 20 mM HEPES (HIMEDIA, India) (pH 7.4), and lysed using a Dounce homogenizer (30–40 strokes on ice). The homogenate was first centrifuged at 800 × *g* for 10 min at 4°C to remove nuclei and unbroken cells. The resulting post-nuclear supernatant (PNS) was centrifuged at 10,000 × *g* for 20 min to eliminate heavy organelles. The supernatant was then ultracentrifuged (Sorvall ultra series centrifuge, Thermo Scientific, USA) at 100,000 × *g* for 1 h at 4°C (SW-41 Ti rotor, Beckman Coulter, USA) to obtain a crude membrane pellet enriched in endosomal compartments.

The pellet was resuspended in 2.0 M sucrose prepared in 20 mM HEPES buffer (pH 7.4) and layered at the top of a discontinuous sucrose gradient (0.8, 1.0, 1.2, 1.4, and 2.0 M in 20 mM HEPES, pH 7.4), followed by ultracentrifugation at 100,000 × *g* for 8–10 h at 4°C. Fractions were collected from the gradient, and the interface between 1.2 and 1.4 M sucrose, enriched in MVBs, was identified based on the presence of MVB marker protein by immunoblotting. This fraction was further diluted in PBS and pelleted again at 100,000 × *g* for 1 h. The final MVB pellet was resuspended in an appropriate buffer for downstream protein analysis.

### Nanoparticle tracking analysis

For nanoparticle tracking analysis (NTA), extracellular vesicles (EVs) were isolated and resuspended in 1× DPBS (Invitrogen, USA). The sample was diluted (100 times from the stock) appropriately to achieve an optimal particle concentration (1 × 10⁶ to 1 × 10⁹ particles/mL) and loaded into the NanoSight system (Malvern Panalytical, UK). The instrument was calibrated, and measurements were conducted at room temperature with five recordings per sample, each lasting 30 s. The analysis parameters, including the detection threshold and blur settings, were optimized to track particle movement under laser illumination accurately. 1× DPBS (Invitrogen, USA) was used as a blank. Data were processed using NTA software (NanoInsight NS300, providing information on particle size distribution, concentration, and mean vesicle diameter.

### Negative-stain transmission electron microscopy (TEM) imaging for EV visualization

For Neuro2A cells infected with JEV, the thin formvar/carbon film-coated 300-mesh copper EM grids (EMS, USA) were glow-discharged for 1 min using a glow discharger. Purified exosomes were fixed with 1 mL of 2% paraformaldehyde (PFA, Invitrogen, USA) for 5 min. A volume of 5–7 µL of the small vesicle suspension was loaded onto each glow-discharged grid and incubated for 1 min. If the exosome concentration was too high, it was diluted to half or one-fifth of the original concentration. The sample was then immediately stained by applying approximately 20 drops of filtered 1% uranyl acetate solution (EMS, USA) onto the grid surface using a syringe. Excess UA solution was gently removed by contacting the edge of the grid with Whatman filter paper. The grid was then quickly rinsed with a drop of distilled water to remove any remaining stain. To dry the sample, the grid was placed on a clean surface and held with tweezers. It was partially covered with a sterile culture dish and left to air-dry for 10 min at room temperature. Once dried, the grid was stored in an EM grid box for subsequent observation under a transmission electron microscope (TEM). For primary cortical neuronal cells infected with JEV, 5 μL of the small vesicle sample was adsorbed onto a glow-discharged 300 mesh carbon-coated Cu grid (EMS, USA) for 90 s. The grid was blotted with Whatman filter paper and washed twice with nuclease-free water for 5–10 s with intermittent blotting. The grid was then stained with one drop of 1% phosphotungstic acid for 45 s and blotted again to remove the residual stain. The grids were air-dried for 15 to 20 min and imaged at room temperature using a JEM1400 transmission electron microscope operated at 120 kV (JEOL) equipped with a LaB6 filament electron gun and a CCD detector at the Advanced Technology Platform Centre (ATPC), Regional Centre for Biotechnology facility, Faridabad, India. The images were recorded at 80K magnification using a low-dose procedure with a pixel size of 6.6 Å/pixel. The raw micrographs were visualized in EMAN2.9 software (63).

### Immunostaining

Twelve-millimeter coverslips were placed in 24-well plates, and cells were seeded at a density of 0.04 × 10⁶ cells per well and incubated for 24 h before treatment or inoculation. Following treatment or infection, cells were washed three times with 1× PBS, then fixed using 4% paraformaldehyde (Invitrogen, USA) for 15 min at RT. The cells were then washed with 1× PBS for 5 min at room temperature and permeabilized with 0.1% Triton X-100 (Sigma-Aldrich, Germany) for 5 min at room temperature. The cells were blocked for 1 h at room temperature in blocking buffer containing 5% FBS or BSA (HIMEDIA, India), 0.05% Triton X-100 (Sigma-Aldrich, Germany), and 0.2 M glycine (BioRad, USA). Cells were incubated at room temperature with the appropriate primary antibody for 1 h in a humidified chamber. Then, cells were washed three times with wash buffer at 5-minute intervals. Corresponding secondary antibodies were added and incubated for 1 h at room temperature in the dark in a humidified chamber. This was followed by washing with 1× PBS three times. Finally, coverslips were mounted using FluorShield Mounting Medium (Abcam, UK), sealed, and stored for immunostaining analysis using a Leica confocal microscope (Leica, Germany). All the images were analyzed by LasX software.

For BODIPY/Lipid spot staining, following inoculation or treatment, cells were washed with 1× PBS and incubated with 2 µM BODIPY (493/503) or with 1× LipidSpot (Thermo Scientific, USA) dye at 37°C for 30 min. After incubation, cells were washed thoroughly with 1× PBS and subsequently fixed for conventional immunostaining procedures.

### Immunohistochemistry of mouse brain cryosections

Mouse brains were harvested following infection and fixed in 4% PFA at 4°C overnight. Fixed tissues were washed with PBS, cryoprotected in 30% sucrose in PBS until fully equilibrated, embedded in Shandon Cryomatrix (Thermo Scientific, USA), and frozen at −80°C. Brain tissues were sectioned at a thickness of 24 µm using a Slee MEV cryostat and mounted onto glass slides.

For immunohistochemistry, mouse brain cryosections were fixed with 4% PFA for 20–25 min at room temperature, followed by three washes with PBS. Sections were then permeabilized with 0.4% PBST for 30 min and washed three times with 0.1% PBST for 5 min each.

LDs were stained using BODIPY 493/503 for 15–20 min, followed by PBS washes. Sections were then blocked in 4% BSA for 1 h at room temperature and incubated overnight at 4°C with primary antibodies against JEV E protein diluted in blocking buffer in a moist chamber.

After washing, sections were incubated with appropriate fluorophore-conjugated secondary antibodies for 1 h at room temperature in the dark. Sections were then washed and mounted using an antifade mounting medium containing DAPI for nuclear staining.

Images were acquired using a confocal microscope under identical settings across all experimental groups. Quantitative analysis of LD accumulation in JEV E protein–positive cells was performed using ImageJ/Fiji software.

### MTT assay

For MTT assay, 0.1 × 10^5^ cells were cultured in 96-well plates for 24 h before respective treatment in triplicate per group per experiment. At specific time points, cells were incubated with freshly prepared 3-(4,5-dimethylthiazol-2-yl)-2,5-diphenyltetrazolium bromide (MTT) (stock concentration 5 mg/mL) (Sigma-Aldrich, Germany) at a final concentration of 0.5 mg/mL at 37°C in the dark for 3–4 h. After incubation, the medium was aspirated, and 100 µL DMSO was added to each well. The formation of formazan was measured in a multimode microplate reader (Multiskan Skyhigh, Thermo Scientific, USA) at an absorbance of 570 nm. All the observed results were compared with the vehicle controls. The percentage of cytotoxicity was calculated with respect to the control wells by using the following equation: % Cytotoxicity = (Control − Test)/Control × 100.

To evaluate the cytotoxicity of GW4869 (MCE, USA), Brefeldin A (BFA) (MCE, USA), an MTT assay was conducted. Cells (1 × 10⁴/well) were seeded in 96-well plates and treated with different doses of GW4869 (1 μM–100 μM) and BFA (1.5–80 μg/mL) for 24 h. After treatment, 20 μL of MTT solution (5 mg/mL, Sigma-Aldrich, Germany) was added and incubated at 37°C for 4 h. Formazan crystals were dissolved in 150 μL of DMSO for absorbance measurement.

### nSmase2 activity assay

Cells were infected as indicated and treated with GW4869 (10 µM or 20 µM) for 12 h or with vehicle control (DMSO). Neutral sphingomyelinase activity was measured using a colorimetric sphingomyelinase assay kit (Abcam, UK) according to the manufacturer’s instructions. Cells were lysed, and equal amounts of protein (20 µg per reaction), quantified using the Bradford protein assay, were incubated with sphingomyelin substrate at 37°C for 2 h. Absorbance was measured at 655 nm. Enzyme activity was calculated from a sphingomyelinase standard curve ranging from 0.07 to 5 mU/mL. All samples were analyzed in duplicate or triplicate. Percentage inhibition of nSMase2 activity was calculated using the formula: % inhibition = [(JEV control − GW4869-treated) / JEV control] × 100.

### Lipid droplet isolation

LDs were isolated from cultured cells using sucrose density gradient ultracentrifugation. Cells were washed with ice-cold PBS, harvested by scraping, pelleted at 1,000 × *g* for 10 min at 4°C, and resuspended in hypotonic lysis medium (20 mM HEPES, 1 mM EDTA, pH 7.4, with protease inhibitors). After incubation on ice, cells were homogenized with a Dounce homogenizer (10–20 strokes) and centrifuged at 1,000 × *g* for 10 min at 4°C to obtain the post-nuclear supernatant. The lysate was adjusted to 20% sucrose, loaded at the bottom of an ultracentrifuge tube, overlaid with 5% sucrose and sucrose-free buffer, and centrifuged at 28,000 × *g* for 30 min at 4°C (SW41Ti rotor; Beckman Coulter). LDs were collected from the top of the gradient for downstream analyses.

### Lipid droplet quantification by flow cytometry

To assess LD accumulation during JEV infection, Neuro2a cells were cultured in 6-well plates at a density of 0.3 × 10^6^ and inoculated with JEV at 5 MOI at 2, 6, 14, 18, and 24 h post-infection (hpi). Cells were harvested by trypsinization, followed by centrifugation at 2,000 × *g* for 1 min. The cell pellets were then resuspended and stained with 2 µM BODIPY 493/503 (Thermo Scientific, USA), a fluorescent neutral lipid dye, for LD detection. After staining, cells were washed with 1× PBS and analyzed using a flow cytometer (BD LSRFortessa Cell Analyzer, BD Biosciences, USA) to quantify LD content at each time point.

### RNA extraction and quantitative reverse transcription-PCR

Total RNA was isolated from cell lysates, extracellular media (EM), and EVs. For cellular RNA extraction, the conventional TRIzol method (Life Technologies, USA) was used according to the manufacturer’s instructions.

RNA from EM and sEV samples was isolated using the Zymo Quick-RNA Micro-Prep Kit (Zymo Research, USA). Briefly, samples were lysed by adding lysis buffer in a 1:1 ratio as instructed in the kit protocol, followed by the addition of ethanol in a 1:1 ratio at room temperature (RT). The mixture was gently mixed and transferred to a Zymo-Spin IC column placed in a collection tube, and centrifuged at 16,000 × *g* for 30 s at RT. The flow-through was discarded. To eliminate genomic DNA contamination, DNase I treatment was performed for each sample as per the kit protocol. Following DNase treatment, 400 µL of RNA Prep buffer was added to the column and centrifuged at 16,000 × *g* for 30 s at RT. The flow-through was discarded. Next, 700 µL of RNA wash buffer was added and centrifuged at 16,000 × *g* for 30 s at RT, followed by a second wash using 400 µL of RNA wash buffer under the same conditions. The column was then transferred to a clean, nuclease-free tube. To elute RNA, 15 µL of DNase/RNase-free water was added directly onto the column matrix and centrifuged at 16,000 × *g* for 30 s at RT. RNA purity and concentration were assessed using a NanoDrop spectrophotometer (Multiskan Skyhigh, Thermo Scientific, USA).

Complementary DNA (cDNA) was synthesized using the iScript cDNA Synthesis Kit (Bio-Rad, USA), following the manufacturer’s instructions. Quantitative real-time PCR (qRT-PCR) was performed using SYBR Green Supermix (Bio-Rad, USA) on the Bio-Rad CFX Maestro system (Bio-Rad USA). GAPDH and β-actin were served as internal controls. The list of primers used is provided in Table 1.

**TABLE 1:** Primers

Gene	Forward primer	Reverse primer
JEV NS3	TTGACAATCATGGCAACG	CCCAACTTGCGCTGAATAA
GAPDH	GCCCTTCTGCCGATGC	CTTTCCAGAGGGGCCATCC
IL-6	CATGTTCTCTGGGAAATCGTG	TCCAGTTTGGTAGCATCCATC
CCL5	TGCCCACGTCAAGGAGTATTTC	AACCCACTTCTTCTCTGGGTTG
IL-18	TCAAAGTGCCAGTGAACC	GTTCTTACAGGAGAGGGTAGA
IFN-β	AAGAGTTACACTGTCTTTGCTATC	CACTGTCTGCTGGTGGAGTTCA
TNF-α	TGTCTGGGCAACCCTTATTC	GCCCATTAGCCCACTTCTT
JEV amplicon	TTGACAATCATGGCAAACGACAAACCAACATTGGACGTCCGCATGAT TAACATCGAAGCTAGCCAACTTGCTGAGGTCAGAAGTTACTGCTATCATGCTTCAGT CACTGACATCTCGACGGTGGCTCGGTGCCCCACGACTGGAGAAGCTCACAACGAG AAGCGAGCTGATAGTAGCTATGTGTGCAAACAAGGCTTCACTGATCGTGGGTGGGG CAACGGATGTGGACTTTTCGGGAAGGGAAGCATTGACACATGTGCAAAATTCTCCTG CACCAGCAAAGCGATTGGAAGAACAATCCAGCCAGAAAACATCAAATACGAAGTTGG CATTTTTGTGCATGGAACCACCACTTCGGAAAACCATGGGAATTATTCAGCGCAAGTT GGG	

### Protein extraction and Western blotting

Cells were seeded at the desired density 24 h prior to viral inoculation or treatment. At the indicated time points, cells were harvested, and total protein was extracted using RIPA buffer along with protease inhibitor (Sigma, USA). Briefly, cells were washed twice with 1× PBS at room temperature, followed by the addition of RIPA buffer (Thermo Scientific, USA). The cells were gently scraped and lysed by repeated vortexing, then centrifuged at 14,000 rpm for 15 min at 4°C. The resulting supernatant was collected and either used immediately or stored for downstream applications. The protein samples were estimated by the Bradford (Sigma, USA) assay.

For Western blotting, protein samples were denatured at 95°C for 10 min and resolved by SDS-PAGE (Mini Protean Tetra System, Bio-Rad, USA). The proteins were then transferred onto a nitrocellulose membrane (Bio-Rad, USA) through semi-dry transfer (Power Blotter, Thermo Scientific, USA) and probed with the following primary antibodies: anti-CD81 (Invitrogen, USA), anti-Alix (Abcam, UK), anti-JEV-NS3 (GeneTex, USA), anti-JEV-capsid (GeneTex, USA), anti-JEV-envelope (GeneTex, USA), anti-perilipin (Proteintech, USA), anti-β-actin (Bioss Antibodies, USA), anti-nSMase2 (GeneTex, USA), anti-HSP90 (CST, USA), and anti-ceremide kinase (Novas Biologicals, USA). All the antibody dilutions were prepared in 3% BSA (HIMEDIA, India) in 1× TBST or 5% skimmed milk (HIMEDIA, India) in 1× TBST. A goat anti-rabbit IgG–conjugated secondary antibody (Bioss Antibodies, USA) and a goat anti-mouse IgG–conjugated secondary antibody (Bioss Antibodies, USA) were used for detection. Protein bands were visualized using enhanced chemiluminescence (ECL) reagents (Thermo Scientific, USA) and imaged using a chemiluminescence detection system (Invitrogen, USA).

To precipitate LD-associated proteins, trichloroacetic acid (HIMEDIA, India) was added to the LD fraction to a final concentration of 10%, and the mixture was incubated on ice for 30 min. The precipitated proteins were collected by centrifugation at 14,000  ×  *g* for 15 min at 4°C, washed twice with ice-cold acetone, and air-dried. The final protein pellet was resuspended in Laemmli sample buffer (Invitrogen, USA) and subjected to SDS-PAGE for downstream analysis.

### Antibody neutralization assay

Neuro2a cells were infected with JEV and treated with a DGAT1 + 2 inhibitor for 2 h. At 18 h post-infection, culture supernatants were collected and incubated with anti-JEV envelope (E) protein neutralizing antibody (25 µg/mL) for 1 h at 37°C to neutralize free virions. Antibody-treated supernatants, DGAT1 + 2 inhibitor-treated supernatants, and untreated JEV-infected supernatants were inoculated into Neuro2a cells for 24 h. Viral RNA was then extracted, and JEV genome copy numbers were quantified by quantitative RT-PCR (qRT-PCR). Viral genomes resistant to antibody neutralization were considered EV-associated virus.

### Generation of plasmid constructs

ElectroMax DH5α-E electrocompetent *E. coli* cells (Invitrogen, USA) were transformed with shRNA constructs targeting four distinct regions of the Smpd3 gene (TR511378A, TR511378B, TR511378C, TR511378D; OriGene, USA) or with a scrambled negative control shRNA (TR30012). For overexpression analysis, Smpd3 overexpression plasmid (NM_0214991; OriGene, USA), along with a control vector plasmid, was transfected into Neuro2a cells. Briefly, 20 µL of electrocompetent cells were mixed with 1 µL of plasmid DNA in a 0.1 cm gap pre-chilled cuvette and electroporated using the following settings: 2 kV, 200 Ω, and 25 µF in Gene Pulser Xcell electroporator (Bio-Rad, USA). Immediately after electroporation, 1 mL of S.O.C. (Invitrogen, USA) medium was added, and the mixture was transferred to a 15 mL culture tube and incubated at 37 °C with shaking at 225 rpm for 1 h. For transformation efficiency control, cells were also transformed with pUC19 plasmid DNA, diluted 1:100 in S.O.C. medium (Invitrogen, USA). Subsequently, 50 µL of the transformed culture was plated onto pre-warmed LB agar (HIMEDIA, India) plates containing 100 µg/mL ampicillin (HIMEDIA, India). Single colonies were picked and cultured in Tartoff-Hobbs Broth (HIMEDIA, India). Plasmid DNA was extracted using the alkaline lysis method by following the manufacturer’s protocol (Helix Biosciences, India) and stored at –80°C for future use.

### Mammalian transfection

Neuro2a cells were cultured in 100 mm culture plates. On the day of electroporation, the cells were trypsinized (0.25% Trypsin-EDTA, Gibco, USA) and resuspended at a density of 1  ×  10⁶ cells in 100 µL of 1× Opti-MEM medium (Gibco, USA). Plasmid DNA was added to the cell suspension at a final concentration of 5 µg/mL. The mixture was then transferred to a 0.1 mL pre-chilled electroporation cuvette. Electroporation was performed under the following conditions: pulse = 2, voltage = 150 V. Immediately after electroporation, the cells were transferred to a culture plate using 0.5 mL of complete media. The plate was gently rocked to ensure uniform distribution of the cells and then incubated at 37°C in a humidified incubator with 5% CO₂.

Neuro2a cells (0.1 × 10⁶ cells/well) were seeded in 12-well plates 24 h before electroporation. Cells were electroporated with Poly I:C (5 µg/mL; MCE, USA) in Opti-MEM using 160 V, 15 ms pulse length, and two pulses. At 24 h post-electroporation, the medium was replaced with complete medium. Protein lysates and culture supernatants were collected at indicated time points. sEV release was quantified by nanoparticle tracking analysis (NTA). For LD analysis, cells were stained with BODIPY and quantified by flow cytometry.

For siRNA knockdown, cells were electroporated with siRNA targeting Seipin (Santa Cruz Biotechnology, USA) (50 nM) or VPS4B (100–200 nM) (Dharmacon, USA) in Opti-MEM, using the same electroporation settings. Cells were transferred to complete medium after electroporation and incubated for downstream analyses. Knockdown efficiency was confirmed by immunoblotting.

### Statistical analysis

All data were evaluated using GraphPad Prism 10.5. The quantified values are expressed as the mean ± standard error of the mean (SEM) for both the *in vitro* and *in vivo* experiments. Statistical significance was analyzed using Student’s *t*-test for pairwise and one-way ANOVA with multiple comparisons for comparing multiple data sets. *P* < 0.5 was considered statistically significant and is denoted in the corresponding figures and legends.

All reagents and instruments used are listed in Table 2.

**TABLE 2:** List of consumables, apparatus, and software

Product	Catalog no., model, or use	Company	Country of origin
Reagents			
Acrylamide-bisacrylamide	67394	SRL	India
Antibiotic-antimycotic solution	15240-062	Gibco	USA
Apo-transferrin	T1428	Sigma-Aldrich	Germany
APS	65553	SRL	India
B27 supplement	17504-044	Thermo Scientific	USA
Bradford	B6916	Sigma	USA
Brefeldin	HY-16592	MCE	USA
BSA	MB083	HIMEDIA	India
Crystal violet	TCS10	HIMEDIA	India
DGAT1+2 inhibitor	A-922500+PF06424439	Cayman	USA
D-glucose	A17A/1616/0206/21	SDFCL	India
DMEM	12100-038	Gibco	USA
DMEM F12	11320-033	Gibco	USA
DMSO, HybridoXL, sterile	TC433	HIMEDIA	India
DPBS	14190-144	Gibco	USA
Density gradient medium (iodixanol)	Ab286850	Abcam	UK
EDTA	43272	SRL	India
ECL reagents	1859700	Thermo Scientific	USA
EM grids	FCF 400-CU	Electron Microscopy Sciences	USA
Ethidium bromide solution	16202	SRL	India
FBS	A525680L	Gibco	USA
Glacial acetic acid	695092	Sigma-Aldrich	Germany
Glycine	MB013	HIMEDIA	India
GW4869	HY-19363	MCE	USA
HEPES	MB106	HIMEDIA	India
Hypotonic lysis buffer	22050012	bioWORLD	India
Insulin	I6654	Sigma-Aldrich	Germany
Laemmli	3016242	Invitrogen	USA
LB agar	M1151	HIMEDIA	India
LB broth	M1245	HIMEDIA	India
MTT	21795	Cayman	USA
2-Mercaptoethanol	M3148	Sigma	USA
Neurobasal medium	211003-049	Thermo Scientific	USA
Nitrocellulose membrane	A30809085	Bio-Rad	USA
Oleic acid	03008	Sigma-Aldrich	Germany
Opti-MEM	31985-062	Gibco	USA
Paraformaldehyde	30525-89-4	Ottokemi	India
4% paraformaldehyde solution	FB002	Invitrogen	USA
Penicillin-streptomycin solution	A002A	HIMEDIA	India
Poly-D-L-lysine	P9155	Sigma-Aldrich	Germany
Polyinosinic-polycytidylic acid	HY-107202	MCE	USA
Ponceau stain	A40000279	Thermo Scientific	USA
Precision Plus Dual Color Protein Standards	1610374	Bio-Rad	USA
Progesterone	P6149	Sigma-Aldrich	Germany
Protease inhibitor	PH340	Sigma-Aldrich	Germany
Putrescine	P5780	Sigma-Aldrich	Germany
Protein A/G magnetic beads	HYK0202	MCE	USA
RNaseZap	AM9780	Invitrogen	USA
RIPA	89901	Thermo Scientific	USA
S.O.C medium	11319-019	Invitrogen	India
Selenium	7782-49-2	Sigma-Aldrich	Germany
Skimmed milk	GRM1254	HIMEDIA	India
Sucrose	27580	SRL	India
SYBR Green Supermix	1725120	Bio-Rad	USA
UltraPure 10× TAE Buffer	15558-042	Invitrogen	USA
Tartoff-Hobbs Broth	M1250	HIMEDIA	India
TCA	GRM6274	HIMEDIA	India
TEMED	MB026	HIMEDIA	India
Tris-base	71033	SRL	India
Triton X-100	T9284	Sigma-Aldrich	Germany
Tween 20	P1379	Sigma-Aldrich	Germany
Trizol	9109	TaKaRa	Japan
Trypan blue stain	15250061	Thermo Scientific	USA
Trypsin-EDTA	25200-072	Gibco	USA
Uranyl acetate	22400-1	EMS	USA
Kits			
High-Yield Plasmid Extraction Mini Kit	HBHPD100	Helix Biosciences	India
iScript cDNA Synthesis Kit	1708890	Bio-Rad	USA
QIAquick Gel Extraction Kit	28704	QIAGEN	Germany
Pierce Gaussia Luciferase Glow Assay Kit	16160	Thermo Scientific	USA
Sphingomyelinase Assay Kit	ab138876	Abcam	UK
Zymo Quick-RNA-Prep kit	R1050	Zymo Research	USA
Antibodies/dyes			
Alix	Ab275377	Abcam	UK
Anti-Mouse Alexa Fluor 488	AB6785	Abcam	UK
Anti-Rabbit Alexa Fluor 555	A21428	Invitrogen	USA
Beta-Actin	BS0061R	Thermo Scientific	USA
BODIPY	D3922	Thermo Scientific	USA
CD81	PA5-11417	Invitrogen	USA
CD63	PA6-92370	Thermo Scientific	USA
Ceramide kinase	NB100-2911SS	Novas Biologicals	USA
Calnexin	66903-1-Ig	Proteintech	USA
ERGIC	16108-1-AP	Proteintech	USA
FluorShield mounting medium	ab104139	Abcam	UK
Goat anti-mouse-HRP	AB205719	Abcam	UK
Goat anti-rabbit-HRP	Bs-0295G	Bioss	USA
Goat anti-mouse-FITC	AB6785	Abcam	UK
HSP90	4874	CST	USA
JEV-capsid	GTX634152	GeneTex	USA
JEV-envelope	GTX125867	GeneTex	USA
JEV-NS3	GTX125868	GeneTex	USA
JEV-prM	GTX131833	GeneTex	USA
Lipid spot	70069-T	Thermo Scientific	USA
nSMase2	GTX135275	GeneTex	USA
Perilipin	15294-1-AP	Proteintech	USA
Seipin	PA5114918	Thermo Scientific	USA
TSG 101	28283-1-AP	Proteintech	USA
TOM20	11802-1-AP	Proteintech	USA
VPS4B	28283-1-AP	Proteintech	USA
VPS4B siRNA	L-044487-00-0005	Dharmacon	USA
Seipin siRNA	Sc-62991	Santa Cruz Biotechnology	USA
Control siRNA	Sc-37007	Santa Cruz Biotechnology	USA
Apparatus			
BIO-RAD CFX Maestro	CFX Opus 196	Bio-Rad	USA
Countess 3	AMQAX2000	Invitrogen	USA
Chemi Doc imaging system	iBright Imaging System	Invitrogen	USA
Confocal microscope	TCS SP8	Leica	Germany
Digital disk tube rotator	GX-Roll-DR24	Genetix Biotech	India
Electroporator	GenePulserXL	Bio-Rad	USA
EVOS XL Core microscope	AMEX1200	Invitrogen	USA
BD LSRFortessa cell analyzer	647797L5	BD Biosciences	USA
Incubator orbital shaker with cooling	BIO-IOS-03	bioWORLD	India
Mini-Protean Tetra System	041BR354097	Bio-Rad	USA
NanoDrop spectrophotometer	Multiskan Skyhigh	Thermo Scientific	USA
Nanoparticle analyzer	NS300	Malvern Pananalytical	UK
PCR machine	C1000 Touch Thermo Cycler	Bio-Rad	USA
Power Blotter XL	PB0010	Invitrogen	USA
Sorvall Ultra Series centrifuge	T865, AH629	Thermo Scientific	USA
Transmission electron microscope (TEM) 120 kV	JEM-1400 Flash	JEOL Ltd.	Japan
SpectraMax M5 Multi-Mode Microplate Reader	SpectraMax M5	Molecular Devices	USA
Slee MEV cryostat	MTP 100-240 V, 50/60 Hz 11000200	SLEE	Germany
Application			
BD FACSDiva	FACS analysis	BD Biosciences	USA
CFX Maestro	qRT-PCR analysis	Bio-Rad	USA
GraphPad Prism version 9.0	Statistical analysis	GraphPad Software, Inc.	USA
Image J	Densitometric and size analysis	National Institutes of Health	USA
LasX	Confocal image analysis	Leica	Germany
NanoInsight	Nanoparticle tracking analysis	Malvern Pananalytical	UK

## Data Availability

All data generated during the research are included in the paper.

## SUPPLEMENTAL MATERIAL

Supplemental figures mbio.00423-26-s0001.pdf Fig. S1 to S8.

ASM does not own the copyrights to Supplemental Material that may be linked to, or accessed through, an article. The authors have granted ASM a non-exclusive, world-wide license to publish the Supplemental Material files. Please contact the corresponding author directly for reuse.
