# Supplementary material for: Lipid droplets and small extracellular vesicles interplay in Japanese encephalitis virus non-lytic release
Source: mBio. 2026 May 20;17(6):e00423-26. doi: 10.1128/mbio.00423-26 (PMC13251402; doi:10.1128/mbio.00423-26)
Supplement: Supplemental figures — Fig. S1 to S8. [file mbio.00423-26-s0001.pdf]

Supplementary Document

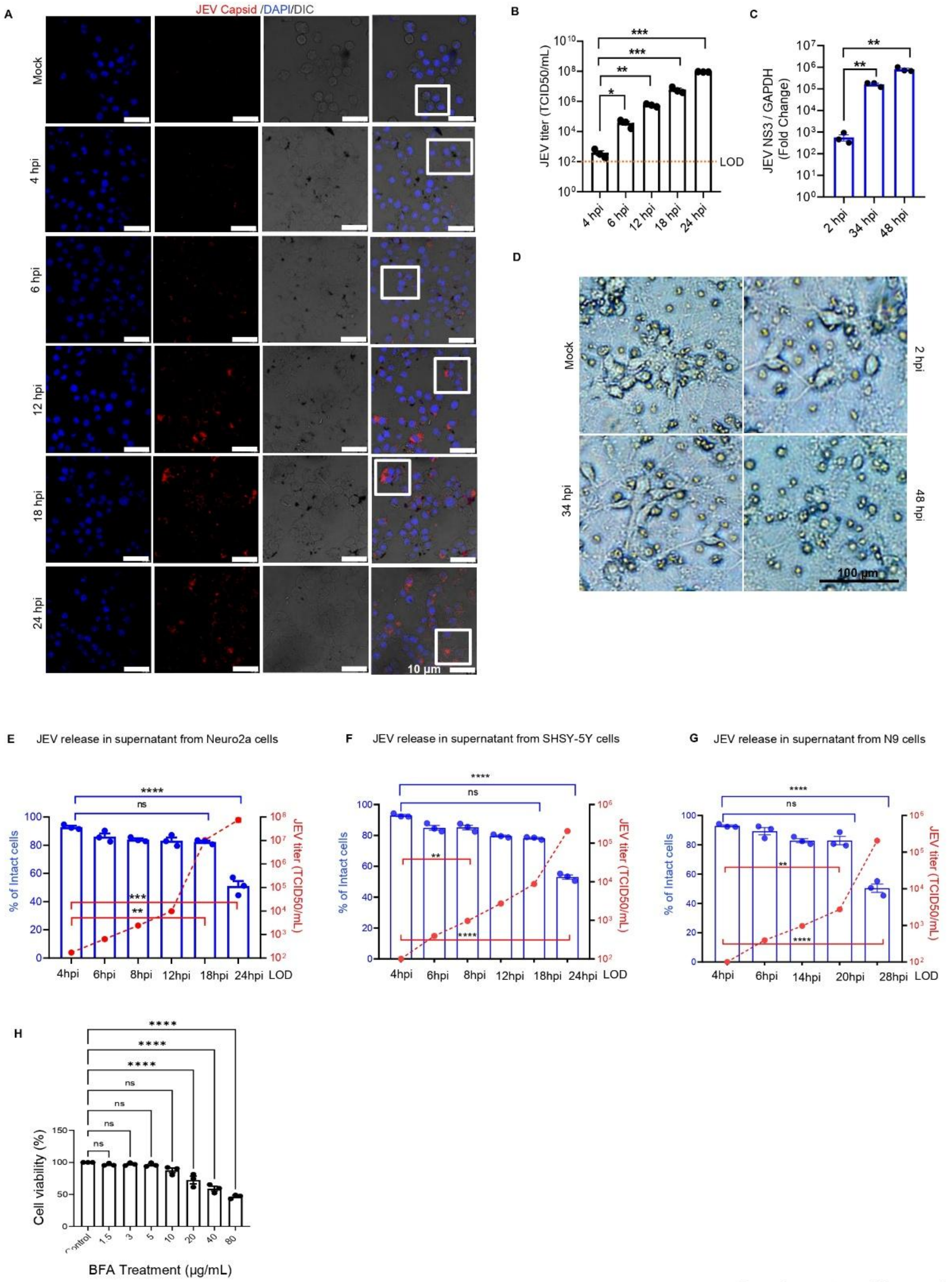

Supplementary Figure. 1

Figure 1. JEV infection in Neuro2a cell line and primary cortical neurons

(A) Representative immunostaining images of Mock- and JEV-infected cells at 4, 6, 12, 18 and 24 hpi. Panels show DAPI-stained nuclei (blue), JEV Capsid protein (red), DIC image, and merged channels. White boxes indicate Capsid-enriched regions. Scale bars 10 µm.

(B) JEV titers in infected Neuro2a cells were measured at 4–24 hpi using a TCID50 /mL assay. Experiments were done in triplicates.

(C) JEV-NS3 mRNA levels in primary cortical neurons were quantified by RT- qPCR at 2 ,34 and 48 hpi. Experiments were done in triplicates.

(D) Representative phase-contrast images of Mock- and JEV-infected primary cortical neurons at the indicated time points. Scale bar, 100 μm.

(E–G) JEV titers in supernatants from Neuro2a (E), SH-SY5Y (F), and N9 (G) cells were measured at the indicated time points by TCID50/mL assay, and cell viability was assessed by Trypan blue staining. Experiments were done in triplicate.

(H) The percentage of Cell Viability was measured by MTT assay at the indicated BFA concentrations. Experiments were done in triplicates

Statistical analysis was performed using one-way ANOVA with multiple comparisons and unpaired t-tests for pairwise comparisons.

*p<0.05, **p < 0.01, ***p < 0.001, ****p < 0.0001, ns= not significant

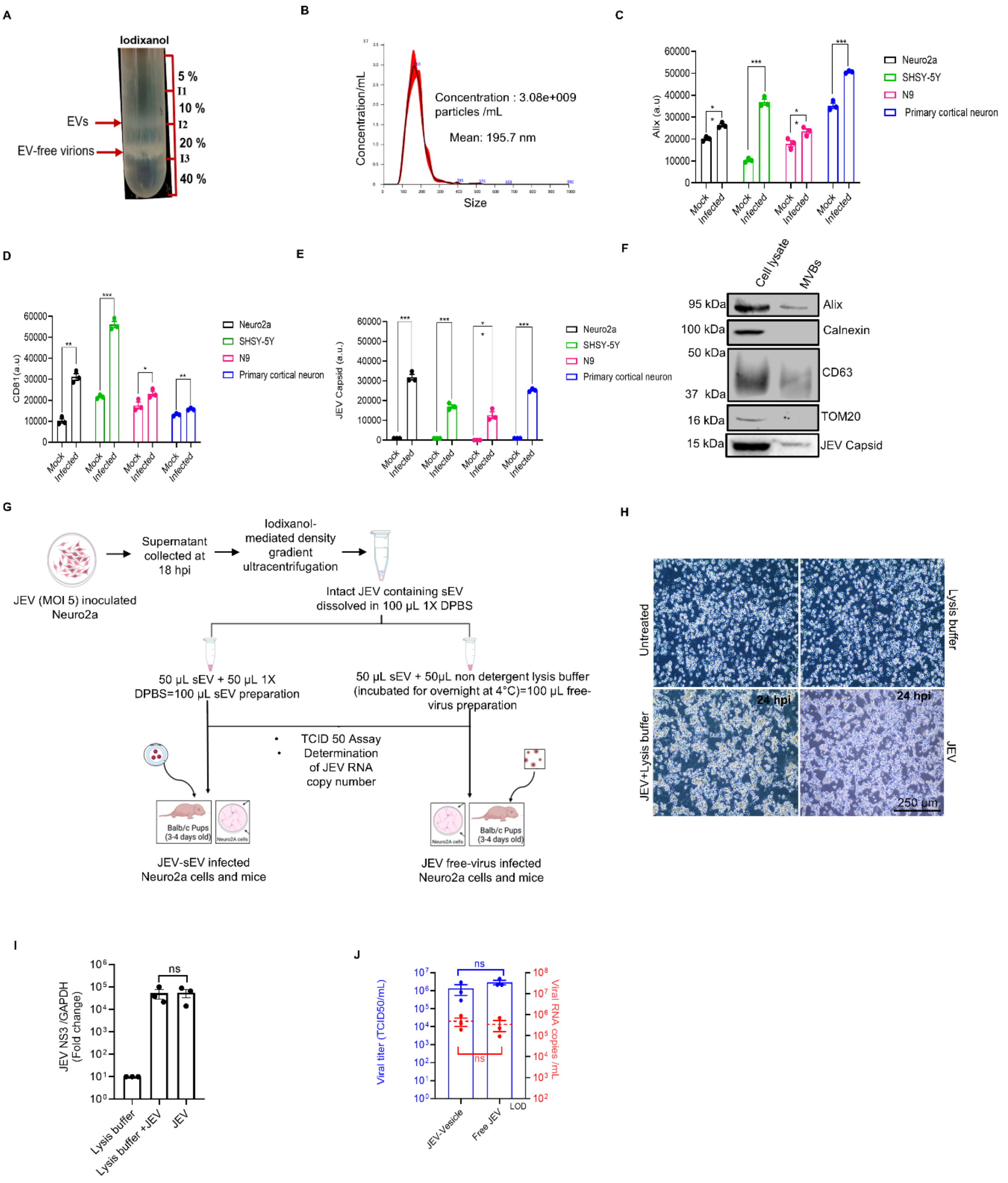

Supplementary Figure. 2

Figure 2. Characterization of EVs in neuronal and microglial cell lines

(A) Representative image of iodixanol gradient fractionation showing the I1, I2, and I3 interfaces separating EVs and EV-free virions.

(B) Representative NTA size distribution profile of CD63+ EVs showing particle concentration and mean vesicle size.

(C–E) Densitometric analysis of Alix (C), CD81 (D), and JEV Capsid protein (E) levels in EVs from different cell types. Experiments were done in triplicates

(F) Representative Western blot showing protein expression of Alix, CD63, and JEV Capsid in JEV-infected (18hpi) cell lysates and isolated MVB fraction, with Calnexin and TOM20 as negative controls to assess cellular contamination. Equal volumes of samples were loaded for normalization.

(G) Schematic of the preparation of EV-associated and EV-free JEV for downstream infectivity and RNA analyses.

(H, I) Representative bright-field images of Neuro2a cells under the indicated conditions (untreated, lysis buffer, JEV + lysis buffer, and JEV) at 24 hpi (H) and JEV NS3 mRNA levels were quantified by RT-qPCR (I).Data represent three independent experiments. Scale bars 250 µm

(J) Viral titers (TCID50/mL) and JEV RNA copies were quantified in JEV-vesicle and Free JEV samples. Data represent three independent experiments.

Statistical analysis was performed using one-way ANOVA with multiple comparisons and unpaired t-tests for pairwise comparisons.

*$p<0.05$, **$p < 0.01$, ***$p < 0.001$, ****$p < 0.0001$, ns= not significant

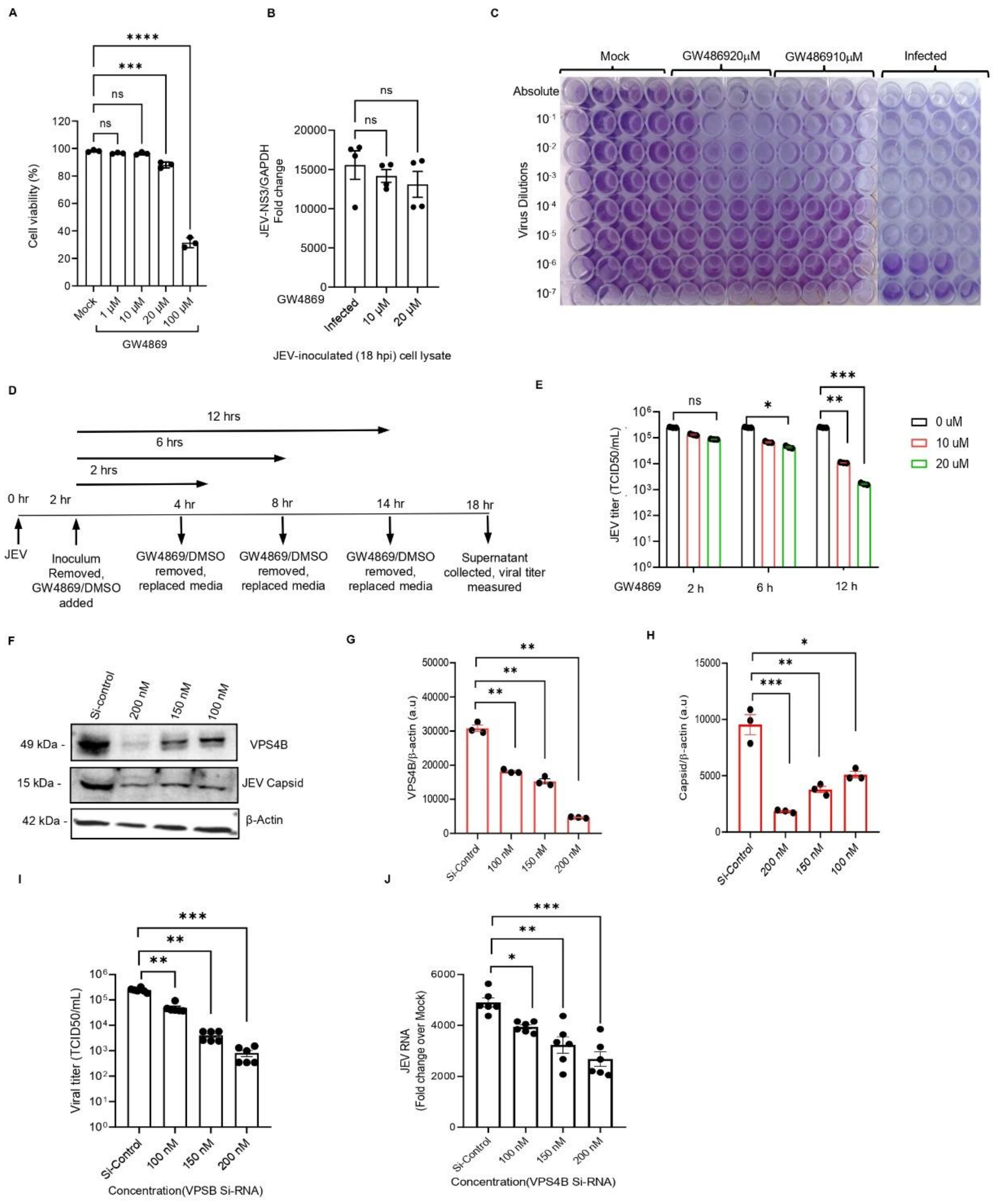

Supplementary Figure. 3

Figure 3. GW4869 treatment and VPS4B knockdown in JEV-inoculated Neuro2a cells

(A) Percentage Cell viability of Neuro2a cells treated with increasing doses of GW4869 was measured by MTT assay. Experiments were done in triplicates

(B) JEV NS3 mRNA levels were quantified by RT-qPCR in JEV-inoculated Neuro2a cells treated with GW4869 (10 or 20 μM) for 12h at 18 hpi. Experiments were in quadruplets.

(C) Representative TCID50 assay plates showing virus titration of Mock, JEV+GW4869-treated (10 and 20 μM), and JEV-infected cells across serial dilutions.

(D) A schematic outline of the GW4869 treatment timeline (2, 6, and 12 h) in JEV-inoculated cells.

(E) JEV titers were measured in the presence of GW4869 at the indicated time points by TCID50/mL Experiments were performed in triplicates.

(F–H) Representative Western blots showing the protein expression of VPS4B and JEV Capsid in cells transfected with a non-targeting control siRNA (Si-control) and increasing concentrations (100nm, 150nm, and 200nM) of VPS4B siRNA and (G,H) their densitometric quantification. β-actin was used as a loading control. Experiments were performed in triplicates. .

(I) JEV titer (TCID50/mL) in the cell culture supernatant after VPS4B siRNA treatment. Experiments were performed 6 times..

(J) Fold change in JEV-NS3 expression determined by RT-qPCR following VPS4B siRNA treatment. Experiments were performed 6 times.

Statistical analysis was performed using one-way ANOVA with multiple comparisons and unpaired t-tests for pairwise comparisons.*$p<0.05$, **$p < 0.01$, ***$p < 0.001$, ****$p < 0.0001$, ns= not significant.

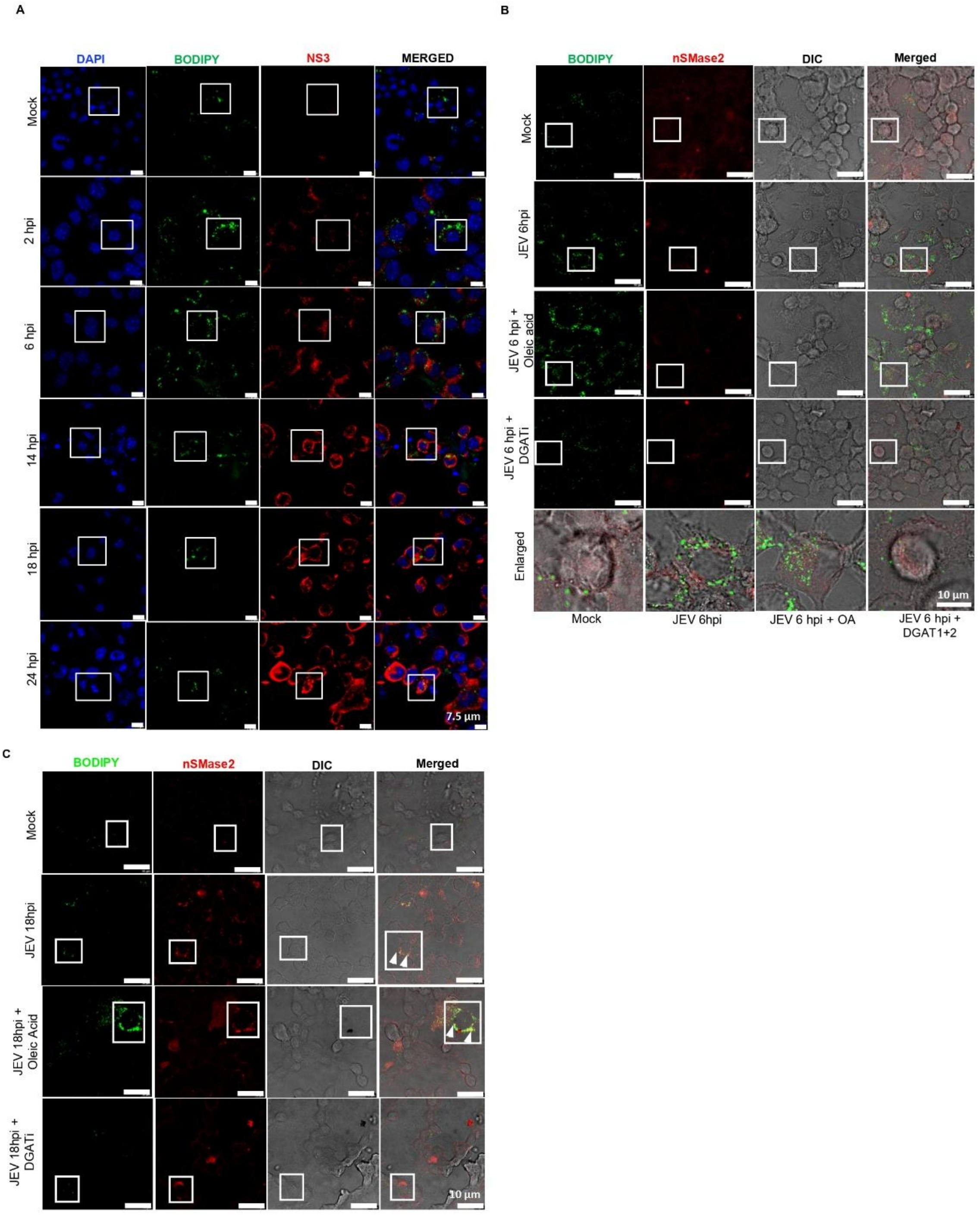

Supplementary Figure: 4

Figure 4. Lipid droplet dynamics in JEV-inoculated Neuro2a cells

(A) Representative immunostaining images showing BODIPY-stained LD and JEV NS3 expression in Mock and JEV (2, 6, 14, 18, and 24 hpi). Scale bar = 7.5 µm. Panels

show DAPI-stained nuclei (blue), Bodipy-stained LD (green) JEV NS3 protein (red), and merged channels.

(B) Representative immunostaining images showing colocalization of LD and nSMase2 under the following conditions: Mock, JEV (6 hpi) + Oleic Acid (OA), JEV infection (6 hpi), and JEV (6 hpi) + DGATi (2h).Scale Bar = 10 μm. Panels show LD (green), nSmase2 (Red), DIC image , and merged channels.

(C) Representative immunostaining images showing colocalization of LD and nSMase2 under the following conditions: Mock, JEV (18 hpi) + Oleic Acid (OA), JEV infection (18 hpi), and JEV (18 hpi) + DGATi (2h).Scale Bar = 10 μm. Panels show LD (green), nSmase2 (Red), DIC image , and merged channels.

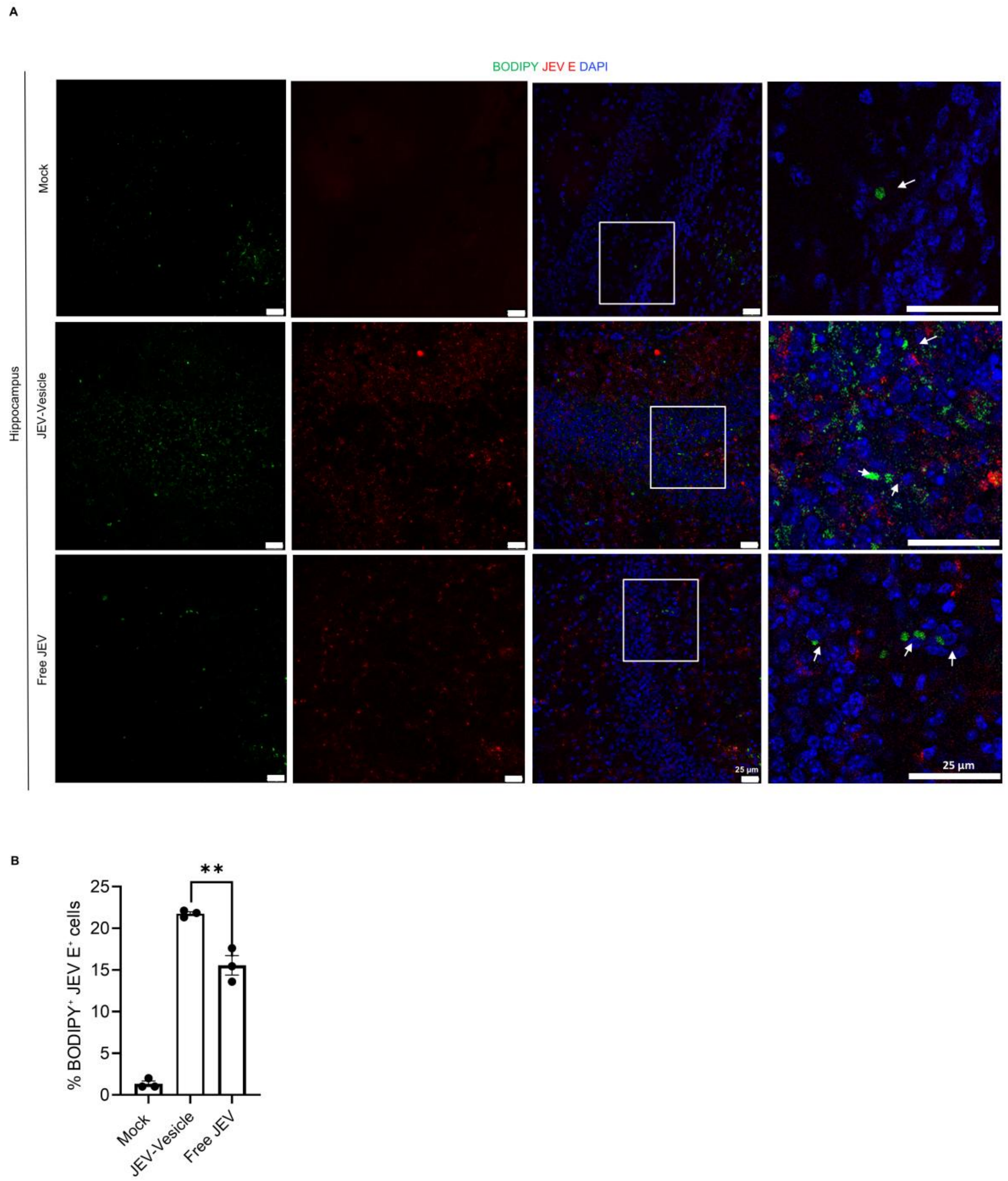

Supplementary Figure. 5

Figure 5. Lipid droplets in Mock, JEV-EVs and Free JEV-infected brain sections

(A) Representative immunostaining images of brain sections from Mock, JEV-Vesicle and Free JEV infected samples showing JEV E protein (red), LD stained with BODIPY (green), and nuclei (DAPI, blue). Arrows indicate cells positive for both JEV and

BODIPY. Scale bar = 25 μm.. Representative images from one of three biologically independent experiments are shown

(B) Quantitative analysis showing the percentage of $BODIPY^{+}$ cells in JEV E^{+} cells across multiple fields.

Statistical analysis was performed using unpaired t-tests for pairwise comparisons. *p<0.05, **p < 0.01, ***p < 0.001, ****p < 0.0001, ns= not significant.

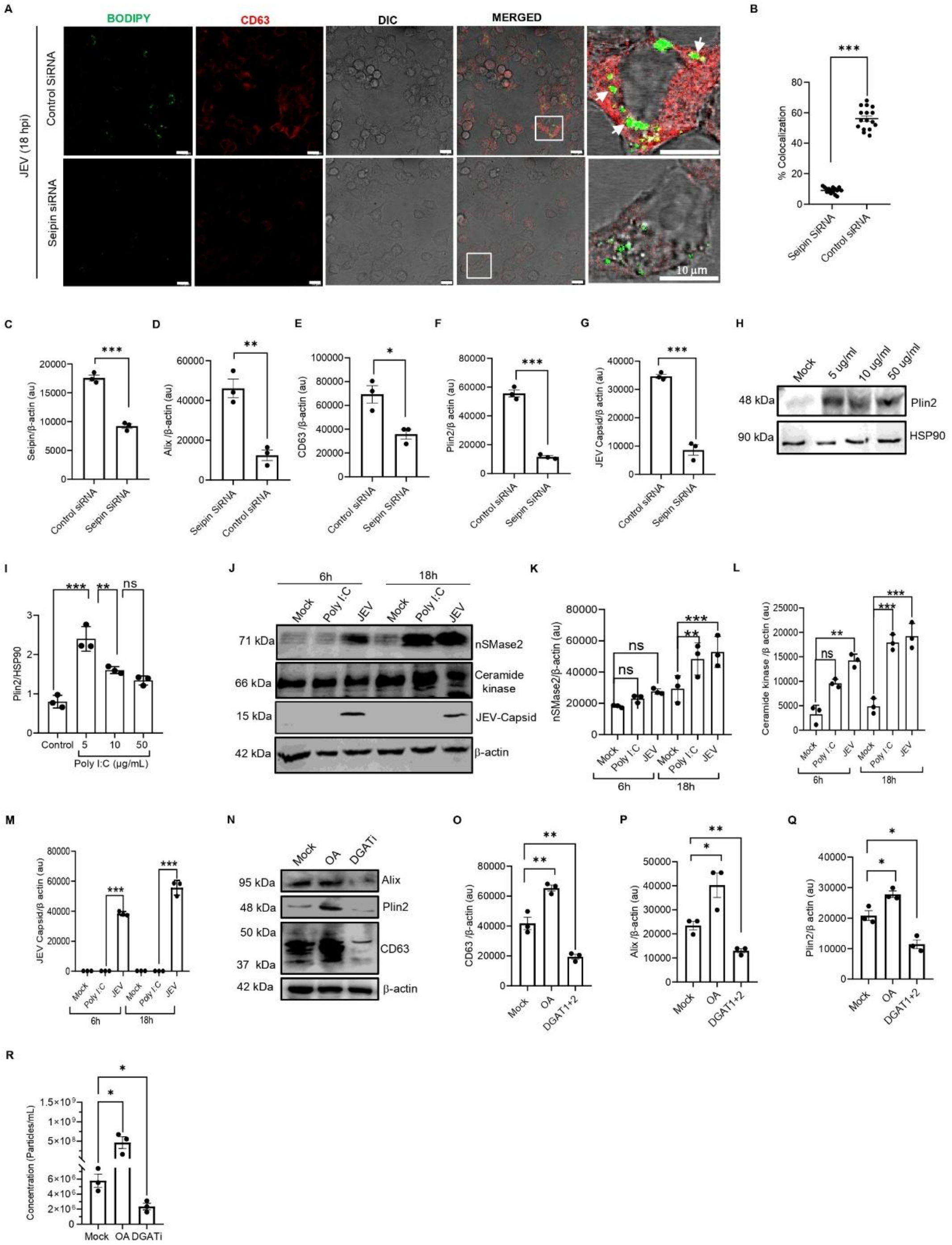

Supplementary Figure. 6

Figure 6. Poly I:C–induced LD dynamics regulate EV biogenesis

(A) Representative immunostaining images showing colocalization of LDs with CD63 in Control siRNA- and Seipin siRNA-treated cells at 18 hpi of JEV infection. Scale bar = 10 µm. Panels display LDs (green), CD63 (red), DIC images, and merged channels. Arrowheads indicate regions of LD–CD63 colocalization. Representative images from one of three biologically independent experiments are shown.

(B) The percentage of colocalization relative to the total number of cells in each field was quantified. Each data point represents the percentage of LD–CD63 colocalization per field.

(C–G) Densitometric quantification of Seipin (C) , Alix (D), CD63 (E), Plin2 (F), and JEV Capsid (G) after Seipin knockdown. Experiments were performed in triplicates.

(H) Representative Western blot showing expression of Plin2 following treatment with varying concentrations of Poly I:C (5, 10, and 50 ug /mL). HSP90 served as the loading control.

(I) Densitometric quantification of Plin2 levels at indicated groups. Experiments were performed in triplicates.

(J) Representative Western blot analysis showing the expression of nSMase2, ceramide kinase, and JEV capsid in mock-treated, Poly I: C-treated (6h and 18 h), and JEV-infected (6h and 18h) cells.

(K-M) Quantification of nSMase2 (K), Ceramide kinase (L), and JEV Capsid (M) expression in Mock, PolyI:C (6h and 18h), and JEV-infected (6h, 18h) cells. Experiments were performed in triplicates.

(N) Representative Western blot showing ALIX, CD63, and PLIN2 expression in Mock-, OA-treated, and DGAT inhibitor-treated cells.

(O-Q) Quantification of CD63 (O), Alix (P), and PLIN2 (Q) expression in Mock, OA-treated, and DGAT inhibitor-treated cells. Experiments were performed in triplicate.

(R) Quantification of EVs concentration by NTA in Mock, OA-treated, and DGAT1+2 inhibitor-treated group. Experiments were performed in triplicates.

Statistical analysis was performed using one-way ANOVA with multiple comparisons and unpaired t-tests for pairwise comparisons.

*p<0.05, **p < 0.01, ***p < 0.001, ****p < 0.0001, ns= not significant.

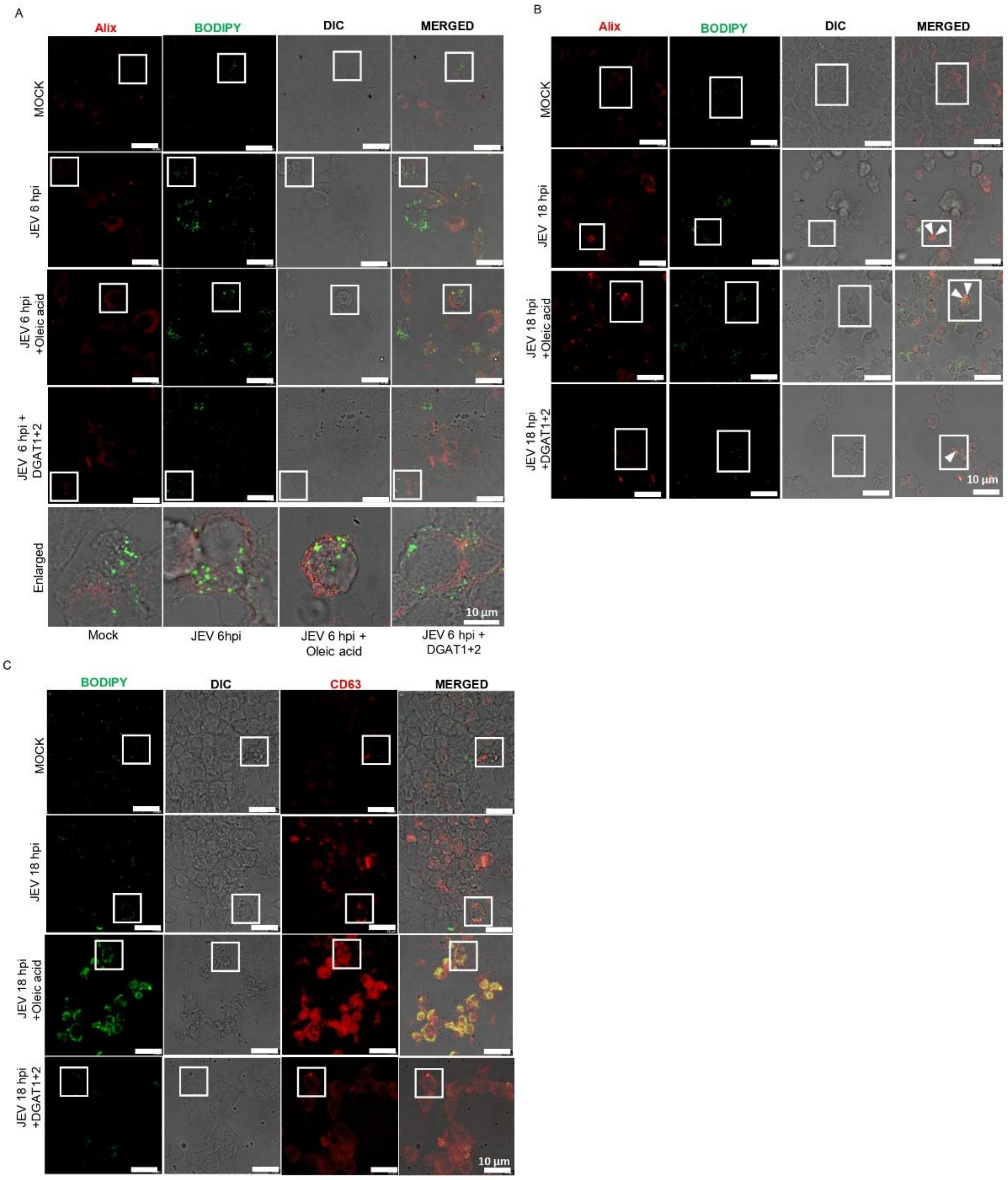

Supplementary Figure .7

Figure 7. LD–MVB interactions regulate sEV-mediated JEV egress

(A,B) Representative immunostaining images showing the colocalization of lipid droplets (LD) with Alix in Neuro2a cells at 6 hpi (A) and 18 hpi (B) at indicated

experimental treatments. Panels show LD (green), Alix (Red) ,DIC image , and merged channels.Scale bar = 10µm

(C) Representative immunostaining images showing colocalization of LD with CD63 in Mock, JEV 18 hpi , JEV 18 hpi +OA, and JEV 18 hpi + DGAT1+2 inhibitors (2 h)–treated Neuro2a cells. Scale bar = 10µm. Panels show LD (green), CD63 (Red) ,DIC image , and merged channels.

Statistical analysis was performed using one-way ANOVA with multiple comparisons and unpaired t-tests for pairwise comparisons.

*$p<0.05$, **$p < 0.01$, ***$p < 0.001$, ****$p < 0.0001$, ns= not significant.

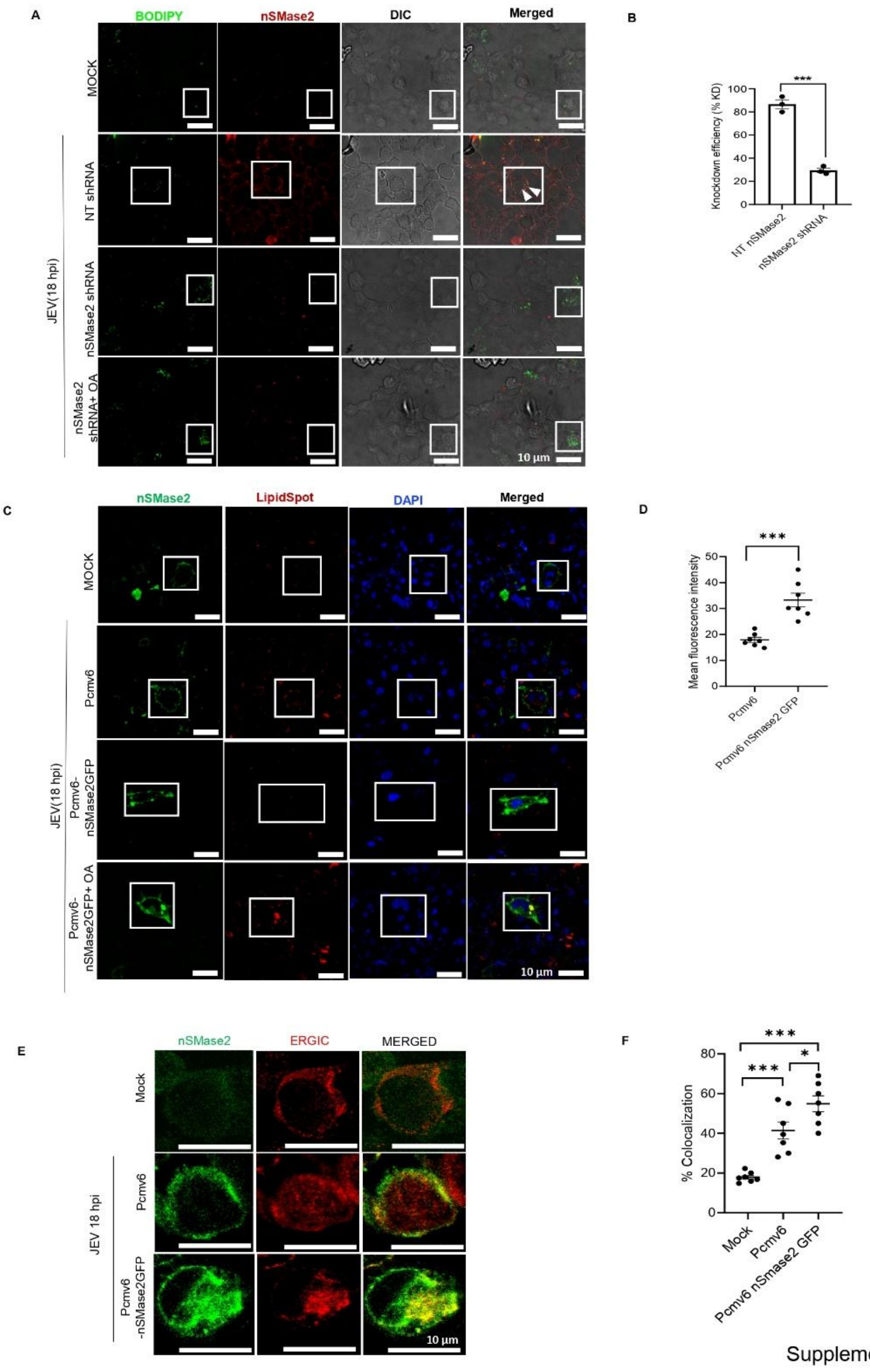

Supplementary Figure. 8

Figure 8. nSMase2 knockdown and overexpression in Neuro2a cells

(A) Representative immunostaining images showing distribution of LD and nSMase2 under the following conditions: Mock, JEV (18 hpi) + NT shRNA, JEV(18 hpi)+ nSMase2 shRNA, and JEV (18 hpi) + nSMase2 shRNA + OA. Scale Bar = 10 µm.. Panels show LD (green), nSmase2 (Red), DIC image, and merged channels.

(B) Percentage of Knockdown efficiency of nSMase2 was quantified relative to control shRNA. Each data point represents the percentage of nSmase2-positive cells per frame.

(C) Representative Immunostaining images illustrating the distribution of LD (LipidSpot staining) and nSMase2 under various conditions: Mock, JEV (18 hpi)+ pCMV6, JEV(18 hpi)+ nSMase2-GFP, and JEV (18 hpi) + nSMase2-GFP + OA. Scale bar = 10 µm.Panels show LD (Red), nSmase2 (Green), DIC image, and merged channels.

(D) MFI quantification of cells transfected with a control vector (Pcmv6) versus overexpressing nSmase2-GFP. Each data point represents the MFI of nSmase2 positive cells per frame.

(E,F) Representative Immunostaining images showing colocalization of nSMase2 with an ERGIC under the indicated conditions. Scale Bar = 10 µm. Panels show nSmase2 (green),ERGIC (Red) and merged channels. Percentage of colocalization quantified (F).Each data point represents the percentage of nSmase2–ERGIC colocalization per cell.

Statistical analysis was performed using one-way ANOVA with multiple comparisons and unpaired t-tests for pairwise comparisons.

$^{*}p<0.05$, $^{**}p < 0.01$, $^{***}p < 0.001$, $^{****}p < 0.0001$, ns= not significant.
